# Supplementary material for: Invasive candidiasis following lung transplant: An Assessment of impact utilizing a national insurance claims cohort
Source: PLoS One. 2025 Aug 21;20(8):e0330162. doi: 10.1371/journal.pone.0330162 (PMC12370120; doi:10.1371/journal.pone.0330162)
Supplement: Attachment — Submitted filename: Reviewed by Seyedeh Elaheh Bagheri.pdf [file pone.0330162.s001.pdf]

Manuscript Number: **PONE-D-25-06214** reviewed by **Seyedeh Elaheh Bagheri**

Overall

I read with great interest the article entitled "Invasive Candidiasis Following Lung Transplant: An Assessment of Impact Utilizing a National Insurance Claims Cohort", which falls within the aim of this journal. This study aimed to assess the incidence, risk factors, and impact of invasive candidiasis on mortality in lung transplant recipients using administrative claims data from individuals enrolled in commercial and Medicare Advantage health plans in the US.

In my honest opinion, the topic and results are interesting for audiences and the paper is almost well structured enough to attract the readers' attention. However, the authors should consider and clarify some points and improve the paper, as suggested below [*: major points, #: minor points].

Abstract section

Please note that the tone of your study's objective in the Introduction part should align with the verb tense used in the Methods and Results parts. Therefore, instead of "This study aims to…", consider using 'This study aimed to….'.

# Please ensure that the final sentence of the Results part clarifies whether the finding is statistically significant. Therefore, include the relevant statistical measures.

* Writing the Conclusion part is crucial, but it is currently insufficient. Additionally, the first sentence is very similar to the first line of the previous part. So please revise it. You may use the following suggestion or a similar one: 'Invasive candidiasis affects approximately 10% of lung transplant recipients and is linked to higher

mortality, prolonged hospitalization, and increased surgical interventions. These findings underscore the importance of early identification and targeted preventive strategies to improve post-transplant outcomes'.

Introduction section

* Please note that the phrase "Since Candida spp. do not have the ability to digest and invade tissues" is not entirely accurate based on various studies, including this one: https://doi.org/10.1016/j.rsma.2023.103258. So kindly revise it.

# Please note that the phrase "its effectiveness has not been established in the lung transplant population" is stated too definitively. It would be preferable to rephrase it as 'evidence exists but is inconclusive'.

Please note that here "One tool that has been utilized to assess clinical incidence and risk factor questions in other clinical domains are large claims databases, such as OptumLabs® Data Warehouse", "One tool" does not align with "are". It is recommended to restructure the sentence. A suggested revision is: 'Large claims databases, such as OptumLabs® Data Warehouse (OLDW), have been widely used to assess disease incidence and risk factors across various clinical domains'.

Methods section

* Regarding your sentence: "We required subjects to have at least 90 days of continuous health plan coverage prior to their lung transplant date and at least 30 days post". Please clarify the rationale behind selecting the 90-day pre-transplant and 30-day post-transplant timeframes.

# In the part Outcomes of Interest, please note that ICD code 112.5 appears to be duplicated.

*	Since your study follows a cohort design, it is preferable not to use the terms “Case” and “Control”, which are more relevant to case-control studies. Please revise these terms throughout the text and figures, replacing them with ‘diseased’ and ‘non-diseased’, respectively. Additionally, when referring to mortality outcomes, please use ‘exposed’ and ‘unexposed’ instead.

#	In the Mortality Analysis part, it would be preferable to briefly explain the rationale behind matching for the selected variables. For instance for confounding variables.

Results section

#	Please ensure consistency in punctuation. In this instance, (619; 48.4%) uses a semicolon, whereas a comma would be more appropriate for consistency.

*	Please note that (Q_1, Q_3) is not equivalent to the interquartile range (IQR), as IQR is calculated as Q_3 minus Q_1. It is recommended to report IQR as a single value in the text while maintaining the (Q_1, Q_3) format in tables for clarity.

#	Regarding the sentence “The most commonly prescribed antifungal were mold-active azoles”, please note that “antifungal” should be written as ‘antifungals’ or, preferably, ‘Antifungal Medications’ to maintain consistency with the terminology used in the tables.

#	In the first paragraph of this section, please remove all instances of (n, x%) as this information is already provided in the table.

#	Please revise the sentence “Of these, 131 (10.2%) LTRs developed IC following LT”. to explicitly refer to ‘lung transplant recipients’ instead of using “Of these”.

*	Preferably, ensure that the variable titles used in the tables are consistent with those in the main text. For example, term like “locally invasive candidiasis/deep-seated candidiasis” in the text are somewhat ambiguous,

whereas the table clearly refers to “Pulmonary Candidiasis”. Please make the necessary revisions and include any additional explanations in parentheses.

# Please clarify what you mean by “IC diagnostic codes are limited”.

* In the Invasive Candidiasis part, please remove all instances of (n, x%) since this information is already provided in the tables. Additionally, mentioning other details is unnecessary; instead, simply state that all had a P-value < 0.05 (also please clarify that if you have considered this threshold in the Statistical Analysis part). Please apply this recommendation to other parts as well.

# For the sentence: “Post-transplant ECMO (OR: 2.34; 95% CI 1.03 to 5.34, p = 0.043) use of greater than 8 days was the only risk factor significantly associated with post-transplant IC on multivariable modeling”. Please remove the information inside the parentheses as previously suggested. Additionally, after “associated with”, add ‘was associated with around 2.3-fold increase in’ to clarify the magnitude of the association.

Please clarify this phrase “No single indication for transplant increased the odds of IC”.

# Please note that the first two sentences in the Effect of Invasive Candidiasis on Mortality part are not appropriate for this part.

* Please ensure that Table 4 is adequately interpreted, as its interpretation is even more important than that of Table 3.

* Please provide appropriate titles for both figures, as they currently lack titles.

# Regarding Table 3, please note that it is unnecessary to include SE and Z values. It is recommended to remove these values from the table.

* Ensure that consistent variable names are used across all tables and carefully review them for uniformity. Additionally, clarify the rationale for including certain variables in Table 4 that are absent from Table 3 (e.g., COPD/Bronchiectasis).

Discussion section

* Please note that this section currently requires further elaboration from literature. It is recommended to include a more detailed comparison of your study with previous research and, if possible, please discuss the reasons for any discrepancies, which could be attributed to for example, different methodologies.

* Regarding the sentence: “We decided to focus on ECMO support as a potential risk factor by setting the index date ...”, please clarify the rationale for selecting ECMO support.

# Please note that the sentence “We cannot glean ECMO configuration placement position from our dataset. It is likely that femoral lines portend a higher risk of IC than ...” presents a claim without sufficient scientific support. It is recommended to revise and integrate the following phrase: ‘Our dataset does not include information on ECMO cannulation sites. While femoral access may increase the risk of IC, further studies are needed to confirm this association’.

* Regarding your sentence: “We found that patients with IC have more than twice the probability of mortality compared to their matched partners”. I did not find the corresponding results in the previous section.

Good *Luck*,

Confidential comments to the editor:

Dear Editor-in-Chief: **PLOS ONE**

In my opinion, the paper is probable to be accepted if all points are employed.

Thank you in advance for your invaluable time and consideration.

Sincerely,

Seyedeh Elaheh Bagheri,

MSc in Epidemiology, Department of Epidemiology and Biostatistics, Faculty of Public Health, Tehran University of Medical Sciences, Tehran, Iran,

BSc in Nurse Anesthesia, School of Paramedicine, Guilan University of Medical Sciences, Langroud, Iran.
