# Supplementary material for: Invasive candidiasis following lung transplant: An Assessment of impact utilizing a national insurance claims cohort
Source: PLoS One. 2025 Aug 21;20(8):e0330162. doi: 10.1371/journal.pone.0330162 (PMC12370120; doi:10.1371/journal.pone.0330162)
Supplement: Attachment — Submitted filename: Re-reviewed by Seyedeh Elaheh Bagheri.pdf [file pone.0330162.s004.pdf]

Manuscript Number: **PONE-D-25-06214R1** re-reviewed by **Seyedeh Elaheh Bagheri**

I sincerely thank the authors for their thoughtful and thorough revisions to the manuscript. Almost all of my previous comments have been addressed. Before resubmitting, I recommend reviewing the revised version without Track Changes to ensure there are no minor issues, such as the presence of two periods at the end of the conclusion sentence in the abstract. Also, please note that the code "112.4" appears twice in the following sentence: "In our population, ... for IC (ICD-9: 112.4, 112.5, 112.8, 112.4, 112.83; ICD-10: 16 B37.1, B37.5, B37.6, B37.7) in ... procedure".

Additionally, I appreciate your attention to comment "*Since your study follows a cohort design, it is preferable not to use the terms "Case" and "Control", which are more relevant to case-control studies. Please revise these terms throughout the text and figures, replacing them with 'diseased' and 'non-diseased', respectively. Additionally, when referring to mortality outcomes, please use 'exposed' and 'unexposed' instead*". However, a few instances of these terms still appear in the text and should preferably be corrected.

Good Luck,

Confidential Comments to the Editorial Team:

Dear Editors: **PLOS ONE**

In my opinion, the paper is probable to be accepted.

Thank you in advance for your invaluable time and consideration.

Wishing you all the best,

Seyedeh Elaheh Bagheri,

Master Student of Epidemiology, Department of Epidemiology and Biostatistics, Faculty of Public Health, Tehran University of Medical Sciences, Tehran, Iran,

BSc in Nurse Anesthesia, School of Paramedicine, Guilan University of Medical Sciences, Langroud, Iran.
