# Supplementary material for: Invasive candidiasis following lung transplant: An Assessment of impact utilizing a national insurance claims cohort
Source: PLoS One. 2025 Aug 21;20(8):e0330162. doi: 10.1371/journal.pone.0330162 (PMC12370120; doi:10.1371/journal.pone.0330162)
Supplement: Attachment — Submitted filename: PONE-D-25-06214_reviewer UPLOAD.docx [file pone.0330162.s002.docx]

PLOS ONE

Invasive Candidiasis Following Lung Transplant: An Assessment of Impact Utilizing a

National Insurance Claims Cohort

--Manuscript Draft--

| Manuscript Number: | PONE-D-25-06214 |
| --- | --- |
| Article Type: | Research Article |
| Full Title: | Invasive Candidiasis Following Lung Transplant: An Assessment of Impact Utilizing a National Insurance Claims Cohort |
| Short Title: | Invasive Candidiasis Following Lung Transplant |
| Corresponding Author: | Kelly Pennington  Mayo Clinic in Rochester  Rochester , UNITED STATES OF AMERICA |
| Keywords: | Lung transplant, Candida, Fungal Infections, Prophylaxis |
| Abstract: | Introduction: Lung transplant recipients (LTRs) are susceptible to invasive candidiasis (IC). This study aims to assess the incidence, risk factors, and impact of IC on mortality in LTRs using a national insurance claims cohort.    Methods: We conducted a retrospective cohort study using administrative claims data from the OptumLabs® Data Warehouse. We identified LTRs from January 1, 2005, to July 31, 2023, using procedural codes. Exclusion criteria included re-transplantations and pre-transplant IC. We employed multivariable logistic regression to identify risk factors for IC and Cox Proportional Hazard models to assess the impact of IC on mortality.    Results: Among 1279 LTRs, 131 (10.2%) developed IC, primarily during the index hospitalization. The median time to IC diagnosis was 32.0 days following transplant. Post-transplant ECMO for more than 8 days was significantly associated with IC (OR:  2.34; 95% CI 1.03 to 5.34). Mortality was significantly higher in LTRs with IC (HR: 2.31; 95% CI 1.45 to 3.67). LTRs with IC had longer hospital stays and more re-operations compared to those without IC.    Conclusion: IC occurs in approximately 10% of LTRs, primarily during the index hospitalization. These findings highlight the need for targeted strategies to prevent IC and manage at-risk LTRs to improve outcomes. |
| Order of Authors: | Kelly Pennington |
|  | Herb Heien |
|  | Hemang Yadav |
|  | Xiaoxi Yao |
|  | Bradley White |
|  | Steve Peters |
|  | Patricio Escalante |
|  | Che Ngufor |
|  | Raymund Razonable |
|  | Cassie Kennedy |
| Additional Information: |  |
| Question | Response |
| Financial Disclosure  Enter a financial disclosure statement that | The author(s) received no specific funding for this work. |

| describes the sources of funding for the work included in this submission. Review the [submission guidelines](http://journals.plos.org/plosone/s/submission-guidelines#loc-financial-disclosure-statement) for detailed requirements. View published research articles from [PLOS ONE](https://journals.plos.org/plosone/) for specific examples.  This statement is required for submission and will appear in the published article if the submission is accepted. Please make sure it is accurate. |  |
| --- | --- |
| Funded studies Enter a statement with the following details: - Initials of the authors who received each award - Grant numbers awarded to each author - The full name of each funder - URL of each funder website - Did the sponsors or funders play any role in the study design, data collection and analysis, decision to publish, or preparation of the manuscript? |  |
| Did you receive funding for this work? |  |
| Competing Interests  Use the instructions below to enter a competing interest statement for this submission. On behalf of all authors, disclose any [competing interests](http://journals.plos.org/plosone/s/competing-interests) that could be perceived to bias this work—acknowledging all financial support and any other relevant financial or nonfinancial competing interests.  This statement is required for submission and will appear in the published article if the submission is accepted. Please make sure it is accurate and that any funding sources listed in your Funding Information later in the submission form are also declared in your Financial Disclosure statement.  View published research articles from [PLOS ONE](http://journals.plos.org/plosone/) for specific examples. | The authors have declared that no competing interests exist. |

| NO authors have competing interests Enter: The authors have declared that no competing interests exist. Authors with competing interests Enter competing interest details beginning with this statement: I have read the journal's policy and the authors of this manuscript have the following competing interests: [insert competing interests here] |  |
| --- | --- |
| * typeset |  |
| Ethics Statement  Enter an ethics statement for this submission. This statement is required if the study involved:   - Human participants - Human specimens or tissue - Vertebrate animals or cephalopods - Vertebrate embryos or tissues - Field research   Write "N/A" if the submission does not require an ethics statement.  General guidance is provided below. Consult the [submission guidelines](http://journals.plos.org/plosone/s/submission-guidelines#loc-guidelines-for-specific-study-types) for detailed instructions. Make sure that all information entered here is included in the Methods section of the manuscript. | This study utilizes de-identified insurance claims data, which does not include direct patient identifiers such as names, dates of birth, or other protected health information as defined by HIPAA. Since all data are pre-existing and de-identified, this research does not involve human subjects as defined by federal regulations (45 CFR 46.102). Additionally, the study does not include any interventions or interactions with individuals, nor does it involve access to identifiable private information.  Per institutional and federal guidelines, research using fully de-identified secondary data is generally considered exempt from IRB review. However, if required, we are happy to provide further documentation to confirm compliance with ethical and regulatory standards. |

Format for specific study types

Human Subject Research (involving human

participants and/or tissue)

Give the name of the institutional review

board or ethics committee that approved the

study

•

Include the approval number and/or a

statement indicating approval of this

research

•

Indicate the form of consent obtained

(

written/oral) or the reason that consent was

not obtained (e.g. the data were analyzed

anonymously)

•

Animal Research (involving vertebrate

animals, embryos or tissues)

Provide the name of the Institutional Animal

Care and Use Committee (IACUC) or other

relevant ethics board that reviewed the

study protocol, and indicate whether they

approved this research or granted a formal

waiver of ethical approval

•

Include an approval number if one was

obtained

•

If the study involved

non-human primates

,

add

additional details

about animal welfare

and steps taken to ameliorate suffering

•

If anesthesia, euthanasia, or any kind of

animal sacrifice is part of the study, include

briefly which substances and/or methods

were applied

•

Field Research

Include the following details if this study

involves the collection of plant, animal, or

other materials from a natural setting:

Field permit number

•

Name of the institution or relevant body that

granted permission

•

Data Availability

Authors are required to make all data

underlying the findings described fully

available, without restriction, and from the

time of publication. PLOS allows rare

exceptions to address legal and ethical

concerns. See the

[PLOS Data Polic](http://journals.plos.org/plosone/s/data-availability)

[y](http://journals.plos.org/plosone/s/data-availability)

and

[FA](http://journals.plos.org/plosone/s/data-availability#loc-faqs-for-data-policy)

[Q](http://journals.plos.org/plosone/s/data-availability#loc-faqs-for-data-policy)

for detailed information.

No - some restrictions will apply

| A Data Availability Statement describing where the data can be found is required at submission. Your answers to this question constitute the Data Availability Statement and will be published in the article, if accepted. |  |
| --- | --- |
| Important: Stating ‘data available on request from the author’ is not sufficient. If your data are only available upon request, select ‘No’ for the first question and explain your exceptional situation in the text box. |  |
| Do the authors confirm that all data underlying the findings described in their manuscript are fully available without restriction? |  |
| Describe where the data may be found in full sentences. If you are copying our sample text, replace any instances of XXX with the appropriate details.   - If the data are held or will be held in a public repository, include URLs, accession numbers or DOIs. If this information will only be available after acceptance, indicate this by ticking the box below. For example: All XXX files   are available from the XXX database (accession number(s) XXX, XXX.).   - If the data are all contained within the manuscript and/or Supporting Information files, enter the following: All relevant data are within the manuscript and its Supporting Information files. - If neither of these applies but you are able to provide details of access elsewhere, with or without limitations, please do so. For example:   Data cannot be shared publicly because of [XXX]. Data are available from the XXX Institutional Data Access / Ethics Committee (contact via XXX) for researchers who meet the criteria for access to confidential data.  The data underlying the results presented in the study are available from (include the name of the third party | Data cannot be shared publicly because of OptumLabs contract. Data are available from the OptumLabs for researchers who meet the criteria for access to confidential data. |

| and contact information or URL).  • This text is appropriate if the data are owned by a third party and authors do not have permission to share the data.  * typeset |  |
| --- | --- |
| Additional data availability information: |  |

Manuscript	[Click here to access/download;Manuscript;candida paper](https://www2.cloud.editorialmanager.com/pone/download.aspx?id=39214929&guid=efc41a4e-2074-4d3f-9429-54323866994b&scheme=1)

[draft.docx](https://www2.cloud.editorialmanager.com/pone/download.aspx?id=39214929&guid=efc41a4e-2074-4d3f-9429-54323866994b&scheme=1)

# Invasive Candidiasis Following Lung Transplant: An Assessment of Impact Utilizing a National Insurance Claims Cohort

Kelly M. Pennington MD^1,2*^, Herb Heien MS^3,4*^, Hemang Yadav MBBS^1,2^, Xiaoxi Yao PhD^3,4,5^, Bradley White^1^,

Steve G. Peters MD^1,2^, Patricio Escalante MD ^1^, Che Ngufor PhD^8^, Raymund R. Razonable MD^2,6^, Cassie C. Kennedy

MD1,2,3

^1^Division of Pulmonary and Critical Care Medicine, Department of Medicine, Mayo Clinic Rochester, MN, USA;

^2^William J. von Liebig Center for Transplantation and Clinical Regeneration, Mayo Clinic Rochester, MN, USA;

^3^Robert D. and Patricia E. Kern Center for the Science of Healthcare Delivery, Mayo Clinic Rochester, MN, USA;

^4^OptumLabs Eden, Eden Prairie, MN, USA; ^5^Department of Cardiovascular Medicine, Mayo Clinic, Rochester, MN; ^6^Division of Infectious Diseases, Department of Medicine, Mayo Clinic Rochester, MN, USA;

*****These authors had equal contributions to the study and manuscript development. They should be considered co-first authors.

**Correspondence to:**

Kelly M. Pennington, MD, Assistant Professor of Medicine, Pulmonary and Critical Care Medicine, Mayo Clinic, 200

First Street SW, Rochester, MN USA 55905; Email: Pennington.Kelly@mayo.edu; Phone 1-507-266-3958; Fax 1507-266-4372

**Key Words:** Lung transplant, Candida, Fungal Infections, Prophylaxis

**Running Title:** Invasive Candidiasis Following Lung Transplant

# Abstract word count: 199/ 350 Manuscript word count: 3543/ 3500

**Abbreviations:**

Chronic Obstructive Pulmonary Disease- COPD

Confidence Interval- CI

Cox Proportional Hazard Model- PH

Current Procedural Terminology- CPT

Cytomegalovirus- CMV

Extra-corporeal Membrane Oxygenation- ECMO

Idiopathic Pulmonary Fibrosis- IPF

International Classification of Diseases, Ninth Revision- ICD-9

International Statistical Classification of Diseases and Related Health Problems, Tenth Revision- ICD-10

Invasive Candidiasis- IC

Invasive Fungal Infection- IFI

Interstitial Lung Disease- ILD

Lung Transplant- LT

Lung Transplant Recipient- LTR

Odds Ratio- OR

OptumLabs® Data Warehouse- OLDW

United States- US

Abstract

**Introduction:** Lung transplant recipients (LTRs) are susceptible to invasive candidiasis (IC). This study aims to assess the incidence, risk factors, and impact of IC on mortality in LTRs using a national insurance claims cohort.

**Methods:** We conducted a retrospective cohort study using administrative claims data from the OptumLabs® Data Warehouse. We identified LTRs from January 1, 2005, to July 31, 2023, using procedural codes. Exclusion criteria included re-transplantations and pre-transplant IC. We employed multivariable logistic regression to identify risk factors for IC and Cox Proportional Hazard models to assess the impact of IC on mortality.

**Results:** Among 1279 LTRs, 131 (10.2%) developed IC, primarily during the index hospitalization. The median time to IC diagnosis was 32.0 days following transplant. Post-transplant ECMO for more than 8 days was significantly associated with IC (OR: 2.34; 95% CI 1.03 to 5.34). Mortality was significantly higher in LTRs with IC (HR: 2.31; 95% CI 1.45 to 3.67). LTRs with IC had longer hospital stays and more re-operations compared to those without IC.

**Conclusion:** IC occurs in approximately 10% of LTRs, primarily during the index hospitalization. These findings highlight the need for targeted strategies to prevent IC and manage at-risk LTRs to improve outcomes.

# INTRODUCTION

Lung transplant recipients (LTRs) are at high risk for invasive fungal infections (IFI), which are linked to an alarming three-fold increase in all-cause mortality(1-3). While *Aspergillus spp.* have traditionally been seen as the primary cause of IFI in LTRs(4-6), more recent single-center studies suggest that invasive candidiasis (IC) may also be an important cause of IFI, particularly in the early post-lung transplant period(7, 8). Since *Candida spp.* do not have the ability to digest and invade tissues, IC typically occurs following a breach in the epithelial barrier such as with surgical incisions or vascular access catheters.

Most lung transplant (LT) centers in the United States (US) have adopted a universal antifungal prophylaxis strategy with triazole antifungals aimed at preventing invasive mold infections(9). Unsurprisingly, topical utilization of nebulized AmBisome without systemic prophylaxis has been associated with breakthrough IC(7). In the absence of universal prophylaxis, the incidence of candidemia at a single Canadian transplant center was reported at 3.5% with most episodes occurring within the first 30 days post-transplant(8). Risk factors for candidemia included pretransplant hospitalization, post-transplant extracorporeal membrane oxygenation (ECMO), and post-transplant renal replacement therapy(8). In non-LT populations, the risk factors for IC and candidemia are clearly established and additionally include gastrointestinal perforation, length of hospital stay, and diabetes mellitus(10).

While universal antifungal prophylaxis may seem like an effective preventative measure for IC, its effectiveness has not been established in the lung transplant population, and antifungal medications can have significant adverse effects, ranging from cardiomyopathy and prolonged QTc to skin cancer, periostitis, and liver dysfunction(11, 12). Triazole antifungal medications are also expensive and interact with immunosuppressive agents, specifically calcineurin inhibitors, which have a narrow therapeutic index necessitating close, serial monitoring.

Given the unclear benefit, potential for toxicity, excessive costs, and drug interactions of antifungal prophylactic medications, it is imperative to have a clear understanding of the true impact of IC and the risk factors associated with IC in LTRs. One tool that has been utilized to assess clinical incidence and risk factor questions in other clinical domains are large claims databases, such as OptumLabs® Data Warehouse (OLDW)(13, 14). The ability to identify a large multiinstitutional cohort of LTRs, control for potential confounders, identify risk factors and diagnostics, all while following the cohort longitudinally makes this approach particularly attractive in attempting to identify the true impact of IC in LTRs.

This study aims to describe the incidence and risk factors associated with IC in LTR and assess mortality among LTRs with IC and without IC using individuals enrolled in commercial and Medicare Advantage health plans in the US.

# METHODS

This study was conducted in accordance with the STROBE (Strengthening the Reporting of Observational Studies in Epidemiology) guidelines. A detailed STROBE checklist has been included as a supplementary document to ensure comprehensive and transparent reporting of the research methods and findings.

## **Data Source**

We conducted a retrospective cohort study using de-identified administrative claims data from OLDW, which includes medical and pharmacy claims and enrollment records for commercial and Medicare Advantage enrollees. This database contains longitudinal health information on enrollees and patients from a diverse mixture of ages, ethnicities and geographical regions across the US(15). OLDW provides real-world outcomes data for LTR in geographically and demographically diverse populations. In accordance with the Health Insurance Portability and Accountability Act, the use of pre-existing, de-identified claims data is exempted from Institutional Review Board review.

## **Study Population**

We extracted all single or bilateral lung transplants occurring in adults (≥ 18 years of age) from January 1, 2005 to

### November 30, 2023 using eligible International Classification of Diseases, Ninth Revision (ICD-9); International

*Statistical Classification of Diseases and Related Health Problems, Tenth Revision* (ICD-10); and Current Procedural Terminology (CPT) codes as previously described(16). We required subjects to have at least 90 days of continuous health plan coverage prior to their lung transplant date and at least 30 days post. Follow-up started after the patients’ index date and continued to the end of enrollment, death, or end of the study period (December 31, 2023).

Re-transplantations were excluded by removing patients who had multiple hospital encounters with LT procedure codes. Patients were required to have a LT stay of at least 5 or more days, and those who had same-day admission and discharge dates were not excluded as they were considered aborted transplant procedures, also known as "dry runs." Patients with IFI prior to their index date were also excluded to help reduce any potential confounding.

***Outcomes of Interest: Risk Factors for Developing Invasive Candidiasis and Its Effect on Mortality*** Our study aimed to evaluate both risk factors of IC, and its effect on mortality.

In our population, IC was defined as any diagnostic code for IC (ICD-9: 112.4, 112.5, 112.8, 112.4, 112.83, 112.5; ICD-10: B37.1, B37.5, B37.6, B37.7) in any position following the LT procedure. These codes could occur during a clinic encounter, emergency department visit, or inpatient stay. Patients with IFI prior to the admission date for LT were excluded from the analysis. Mortality data was sourced primarily from the Social Security Death Master File, with additional information obtained from hospital discharge records to enhance the comprehensiveness of inpatient death records(16, 17).

Independent variables of interest included sex, age, race/ethnicity, census region, Elixhauser comorbidity score(18), single versus double lung transplant, and indication for transplant. High co-morbidity burden was defined as Elixhauser index of ≥4. Baseline factors were extracted during the respective 90-day baseline period from the index date. An algorithm to identify the indication for transplant utilized ICD-9 and ICD-10 codes aimed at the specific chronic respiratory diseases. These codes could be found in any diagnosis position on a claim during the lung transplant stay to classify the most likely indication for transplant(16).

Other factors considered were pre-transplant corticosteroid use, post-transplant re-operation within the lung transplant hospital stay, post-transplant extra-corporeal membrane oxygenation (ECMO), post-transplant renal replacement therapy, and post-transplant cytomegalovirus (CMV) disease. Pre-transplant corticosteroid use was defined as a pharmacy prescription fill for prednisone, dexamethasone, or equivalent within 30 days prior to the lung transplant procedure. Post-transplant re-operation was defined as a procedure code with description of “chest closure”, “chest exploration” or “chest washout” (CPT: 00520, 21750, 35820; ICD-9: 3451, 3452) occurring more than 24 hours after the initial transplant procedure and within the index hospitalization. Post-transplant ECMO support and posttransplant renal replacement therapy were defined as ICD-9 or ICD-10 (ECMO: Z92.81; renal replacement therapy: Z99.2) diagnostic or procedure code within the index hospitalization. Post-transplant CMV disease was defined as the presence of an ICD-9 (771.1) or ICD-10 (B25.9) diagnostic code for CMV disease in any diagnostic position following transplant.

Antifungal medications prescribed within 90 days prior to a diagnostic code for IC were also considered in our analysis. Antifungal medications included caspofungin, micafungin, anidulafungin, fluconazole, itraconazole, posaconazole, voriconazole, isavuconazonium, amphotericin B deoxycholate, and liposomal amphotericin. Nebulized and intravenous amphotericin B deoxycholate and liposomal amphotericin could not be differentiated. In the absence of prior diagnostic codes for IFI, these were assumed to be nebulized.

## **Statistical Analysis**

To protect patient confidentiality and in accordance with OptumLabs® restrictions, any event frequency of 11 or fewer participants was masked. Descriptive statistics were employed to describe the overall cohort. Categorical variables were summarized as frequency (%) and were compared using chi-square test. Continuous variables were expressed as mean ± standard deviation (SD) or median with interquartile range (IQR), as appropriate and were compared using a *t* test. The tables were analyzed across the case and control groups to test for differences.

Analyzing both risk factors and mortality required us to use two different models and extraction methods to gain better insight of risk factors and the effect IC had on patient mortality. For both sets of analysis, we re-extracted patient enrollment and baseline characteristics and analyzed the data using both descriptive and multivariable methods. Each data extraction was anchored on each of the respective index dates, which created two very similar but unique sets of data for analysis. The patient counts appear approximately the same but should be thought of as two different data sets with very similar patient characteristics.

### Risk Factor Analysis

In our first analysis, we considered all LTRs who met the inclusion criteria for our study. We then assessed *a priori* which risk factors were associated with IC. The analysis was complicated by the fact that IC was most likely to occur during the LT stay, thus to ensure that all considered time dependent risk factors were measured prior to IC, we defined the index date as either the inpatient admission date or the first ECMO date after the LT transplant so that the effect of ECMO (a previously described risk factor for IC) could be assessed. We subsequently excluded patients who had IC before either of these dates.

A multivariable logistic regression model was used to investigate which risk factors were associated with IC. The odds ratios (ORs) and 95% confidence intervals (CIs) were computed to estimate the strength of the associations among the baseline risk factors on the outcome of IC. Baseline risk factors were captured either on or prior to the index date.

### Mortality Analysis

For this analysis, we matched one case to two controls on age ±5 years, sex, index date with respect to the LT admission date, and length of hospital stay. We further assessed the covariate balance between the cases and controls by calculating the standardized differences for each of the covariates in the pre and post matched cohort. A commonly accepted cutoff of 0.10 was used as the threshold for imbalance, where values ranging from 0 to < 0.10 indicate negligible imbalance. All imbalanced variables outside of the ± 0.10 range were controlled for in the final Cox Proportional Hazard (PH) model.

For the PH model, the first date of IC was the index date. For patients without IC, we do not have a similar date but instead have an LT admission date as their first entrance date. To adjust for this, we first ran an exhaustive match using the LT admission date as an index date. Next, we reset all the cases’ index date to the first date of IC after the LT admission date. Then a pseudo index date for the control group was set to be equidistant as the cases’ LT admission date to IC, where the minimal difference in the patients’ LT length of stay were given precedence in the matching criteria. This allowed us to line up the matching controls’ index dates with the case’s index date and help minimize potential confounding in timing to mortality. Enrollment was re-evaluated and all baseline characteristics 90 days prior to the newly assigned index date were analyzed. We then completed a PH model along with a Kaplan Meier curve to evaluate the effect of IC on patient mortality between the groups.

All analyses were conducted using SAS Enterprise Guide 7.13 (SAS Institute Inc., Cary, NC) and Stata version 16.0 (Stata Corp).

# RESULTS

We identified a total of 2,879 unique patients with LT codes from January 1, 2005, to November 30, 2023; 1,279 patients met inclusion and exclusion criteria (**Figure 1**). The most common indication for lung transplant was idiopathic pulmonary fibrosis (IPF) (619; 48.4%) followed by non-IPF interstitial lung disease (ILD) (260, 20.3%) and chronic obstructive pulmonary disease (COPD) (241, 18.4%) (**Table 1**). Most patients underwent bilateral lung transplant (972, 76.0%). The median follow-up was 469.0 (IQR: 224.0, 969.0) days. Most LTRs were prescribed antifungal medications (1,194, 93.4%) during their LT admission. The most commonly prescribed antifungal were mold-active azoles (voriconazole, posaconazole, and isavuconazonium) (699, 54.7%) followed by nystatin (317, 24.8%) and narrower spectrum azoles (itraconazole and fluconazole) (117, 9.2%).

## Invasive Candidiasis

Of these, 131 (10.2%) LTRs developed IC following LT. The majority of initial episodes of IC (69, 52.7%) occurred during the transplant hospitalization. Median time from LT admission to a diagnostic code for IC was 32.0 (IQR 0, 192.0) days. Based on the diagnostic billing codes, locally invasive candidiasis/ deep-seated candidiasis (96, 73.3%) was most common followed by candidemia (24, 18.3%). Please note that IC diagnositic codes are limited.

LTRs who developed IC had significantly longer hospital stays on average (26.0 days, IQR: 15.0, 68.0) compared to those without IC (20.0 days, IQR: 13.0, 38.0; *p*<0.0001) (**Table 2**). They were also more likely to have a re-operation (52, 36.7%) compared to those without IC (313, 27.3%; *p*=0.003). Days on ECMO was significantly greater in those with IC (11.0 days, IQR: 9.0, 50.0) compared to those without IC (7.0 days, IQR: 2.0, 15.0; *p*=0.01). Although ECMO use was not significantly different between the groups (IC: 13, 9.9%; no-IC: 90, 7.8%; *p*=0.41).

## Risk Factors for Invasive Candidiasis: Multivariable Analysis

Post-transplant ECMO (OR: 2.34; 95% CI 1.03 to 5.34, *p* = 0.043) use of greater than 8 days was the only risk factor significantly associated with post-transplant IC on multivariable modeling. The presence of antifungal prophylaxis during the 90 days prior to IC (OR: 1.49; 95% CI 0.85 to 2.59, *p*=0.165) did not significantly change the odds of IC. CMV disease (OR: 1.91; 95% CI 0.43 to 8.51, *p* = 0.395), diabetes mellitus (OR: 1.77; 95% CI 0.80 to 3.92, *p*=0.156), high co-morbidity burden (OR: 0.86; 95% CI 0.44 to 1.70, *p*=0.67), bilateral lung transplant (OR: 0.90; 95% CI 0.59 to 1.39, *p*=0.643), and pre-transplant steroid use (OR: 1.12; 95% CI 0.69, 1.83, p=0.641) were not significant risk factors for IC. No single indication for transplant increased the odds of IC (**Table 3**).

## Effect of Invasive Candidiasis on Mortality

We were able to match 126 cases of IC to 252 controls (**Figure 2**). **Table 4** summarizes the cohort and the standardized differences. The median follow-up was 439.0 (IQR: 231.0, 1001.0) days after index date. There were 97 (25.7%) deaths during follow-up. Median time to death was 347.0 (IQR: 94.0, 797.0) days.

All-cause mortality was significantly higher in those who developed IC (event rate per 100 person-years: 11.32; HR: 2.13; 95% CI 1.45 to 3.12, *p*<0.001). This held true when matching IC cases to controls (event rate per 100 personyears: 12.87; HR: 2.31; 95% CI 1.45 to 3.67, *p*<0.001) (**Figure 3**).

# DISCUSSION

Our study utilized administrative claims data to evaluate both risk factors and the impact of IC in a diverse cohort of LTRs. We found that IC occurred in 10.2% of LTRs. This predominantly occurred during the index hospitalization, with median time to IC diagnosis of 32 days. This is similar to the findings of Baker et. al. in their single center cohort where they found the prevalence rate of IC to be 11.4% with median time to IC of 31 days(7). Marinelli et. al.

described a lower prevalence of IC (3.5%) in their single center cohort(8); however, they only looked at episodes of candidemia. Candidemia compromised approximately half of the episodes of IC in Baker et. al.(7). While

administrative claims database gives us the advantage of looking at patients across institutions, we cannot evaluate the granular details of the type of candida infections (i.e. candidemia versus pleural space infections or surgical site infections). Candidemia was the diagnosis code utilized in 18.3% of IC episodes in our cohort.

We found that post-transplant ECMO support of greater than 8 days is a risk factor for IC in LTRs. Post-transplant ECMO support and renal replacement therapy were risk factors reported by Marinelli et. al.(8) and coincides with risk factors reported in other critically ill populations(10, 19). Re-operation has not previously been reported as a risk factor for IC in LTRs. However, it is a known risk factor for IFI in other solid organ transplant recipients, and an open chest has been reported as a potential risk factor for IC in heart transplant recipients(20),(21). In our data, due to both the small sample size and the need to show a clear chronology directionality with potential risk factors, we were not able to fully explore re-operation within the index hospitalization or post-transplant renal replacement therapy as risk factors for IC. While re-operation was significantly higher in the IC group, we cannot be certain if that was a risk factor for or consequence of IC. We saw no significant difference in the incidence of post-transplant renal replacement therapy between the groups. We decided to focus on ECMO support as a potential risk factor by setting the index date to the first date of ECMO among patients with ECMO utilization. This maintained a 90-day washout period of IC and a proper baseline period to collect risk factors of contracting IC post-index date. While the other measures are important, the direction of causality would be less reliable in the model if they were present.

We cannot glean ECMO configuration placement position from our dataset. It is likely that femoral lines portend a higher risk of IC than internal jugular or subclavian lines; however, any breach in the epithelial barrier and prolonged critical illness in immunosuppressed LTRs likely increases the risk of IC.

Absence of antifungal prophylaxis during the index hospitalization was not a risk factor for IC in our cohort, but around 90% of patients were prescribed some type of antifungal medication making it difficult to discern if absence of an antifungal medication would increase the risk of IC.

Traditional risk factors for IC in non-immunosuppressed patients such as diabetes mellitus and co-morbidity burden were not associated with increased risk of IC in our cohort. Likewise, CMV disease has been implicated as a risk factor for IFI(21), but this was not a risk factor for IC in our cohort. These differences are possibly due to the small sample size or possibly because other studies have grouped IC and invasive mold infections as a composite outcome. Pathophysiologically, CMV as a risk factor for invasive mold infections makes sense as it causes direct tissue damage within the lungs weakening alveolar barrier defenses(22).

We found that patients with IC have more than twice the probability of mortality compared to their matched partners. Marinelli et. al. found in their single center cohort study that LTRs with candidemia had an increased 30-day posttransplant mortality(8). A 3-fold increase in mortality in the first year following transplant has also been described in thoracic transplant recipients at Stanford University between 1980 and 2004(23). While we have had significant advancements in antifungal medications since the Stanford University cohort, mortality rates in LTRs with IC remains staggeringly high. Future efforts should be focused on preventative measures for the development of IC in LTRs. This needs to go beyond pharmacologic prophylaxis, as most patients in our cohort were on some type of antifungal medication and the incidence of IC was around 10%.

## Limitations

The primary constraint inherent to any observational study is the potential for uncontrolled confounding variables. In our investigation, due to the absence of pertinent data within the de-identified cohort, we were unable to consider additional risk factors such as intensified immunosuppressive regimens, pre-transplant colonization status, or parenteral nutrition. Nevertheless, we diligently managed known confounding factors by including variables such as sex, age, race/ethnicity, geographical region, year of transplant, primary diagnosis, type of transplant (single versus double or heart/lung), and comorbidities in our analysis.

One notable advantage afforded by the OLDW is the real-world, enriched racial, ethnic, and geographic diversity of the cohort it encompasses, setting it apart from single observational studies. In comparison to frequently used datasets like the Scientific Registry of Transplant Recipients (SRTR), although our lung transplant cohort is relatively smaller, it furnishes additional data not readily available in SRTR. However, it's imperative to acknowledge a drawback associated with this dataset; it is confined to patients enrolled in commercial and Medicare Advantage health plans. This limitation may potentially curtail the generalizability of our study, as it does not encompass uninsured patients or those enrolled in other government health plans.

Furthermore, the inherent nature of the de-identified dataset introduces certain limitations. For instance, our reliance on the Social Security Death Master File for mortality data comes with constraints, notably the limitations of this database since 2011, which may not capture up to one-third of deaths(24). To mitigate this limitation, we supplemented the Social Security Death Master File with information derived from discharge status and insurance discontinuation due to death (insurance knowledge of death), particularly since the majority of early LT deaths occur within the hospital setting. While we acknowledge the possibility of missing a small proportion of patients who may have passed away outside the hospital environment, we anticipate that this phenomenon would be comparable between the groups we are comparing. Lastly, it is important to recognize that we employed billing codes to identify cases of IC. These codes are contingent on accurate diagnosis and coding by the treating healthcare provider, introducing a potential source of variability and error in our analysis.

# CONCLUSION

In our multi-institutional cohort, IC has a prevalence of around 10% in LTRs with most episodes initially occurring during the index hospitalization. Post-transplant ECMO support for greater than 8 days increases the risk of IC, and patients with IC have significantly more re-operations and longer hospital length of stay. LTRs who developed IC had a two-fold increased risk of death.

**Conflict of interest:** None of the authors have any conflict of interest to disclose. All authors have reviewed and contributed to this manuscript.

**Author Contributions:** KMP, SGP, BW, HH, XY, PE, CN, RRR, CCK: developed concept and study design. HH, XY, CN: acquired and evaluated the claims data. KMP and HH: drafted initial manuscript. All authors reviewed and approved final manuscript.

**Financial Support**: KMP is supported by the Mayo Clinic Department of Medicine Catalyst Award, the Lucile

Nelson Benefactor Career Development Award, and William J. von Liebig Center for Transplantation and Clinical

Regeneration Transplant Scholarly Award, Mayo Clinic, Rochester, MN. Supported by NHLBI grant K23HL151671 (H.Y.). Study contents are solely the responsibility of the authors and do not necessarily represent the official views of the NIH.

**Data Availability Statement:** The data that support the findings of this study are available from the OptumLabs® Data Warehouse but restrictions apply to the availability of these data, which were used under license for the current study, and so are not publicly available. Data are however available from the authors upon reasonable request and with permission of OptumLabs®. Any data sharing will adhere to relevant data privacy regulations and will require a data sharing agreement.

**References:**

1. Arthurs SK, Eid AJ, Deziel PJ, Marshall WF, Cassivi SD, Walker RC, et al. The impact of invasive fungal diseases on survival after lung transplantation. Clinical transplantation. 2010;24(3):341-8.
2. Neofytos D, Fishman JA, Horn D, Anaissie E, Chang CH, Olyaei A, et al. Epidemiology and outcome of invasive fungal infections in solid organ transplant recipients. Transplant Infectious Disease. 2010;12(3):220-9.
3. Pappas PG, Alexander BD, Andes DR, Hadley S, Kauffman CA, Freifeld A, et al. Invasive fungal infections among organ transplant recipients: results of the Transplant-Associated Infection Surveillance Network (TRANSNET). Clinical infectious diseases : an official publication of the Infectious Diseases Society of America. 2010;50(8):1101-11. 4.	Chong PP, Kennedy CC, Hathcock MA, Kremers WK, Razonable RR. Epidemiology of invasive fungal infections in lung transplant recipients on long-term azole antifungal prophylaxis. Clinical transplantation. 2015;29(4):311-8. 5.	Hosseini-Moghaddam SM, Ouedraogo A, Naylor KL, Bota SE, Husain S, Nash DM, et al. Incidence and outcomes of invasive fungal infection among solid organ transplant recipients: A population-based cohort study.

Transplant infectious disease : an official journal of the Transplantation Society. 2020;22(2):e13250.

6. Husain S, Silveira FP, Azie N, Franks B, Horn D. Epidemiological features of invasive mold infections among solid organ transplant recipients: PATH Alliance R registry analysis. Medical mycology. 2017;55(3):269-77.
7. Maziarz EK, Workman AD, Perfect JR, Baker AW, Johnson MD, Arnold CJ, et al. Invasive Fungal Infection after Lung Transplantation: Epidemiology in the Setting of Antifungal Prophylaxis. Clinical Infectious Diseases.

2020;70(1):30-9.

8. Marinelli T, Pennington KM, Hamandi B, Donahoe L, Rotstein C, Martinu T, et al. Epidemiology of candidemia in lung transplant recipients and risk factors for candidemia in the early posttransplant period in the absence of universal antifungal prophylaxis. Transpl Infect Dis. 2022;24(2):e13812.
9. Pennington KM, Yost KJ, Escalante P, Razonable RR, Kennedy CC. Antifungal prophylaxis in lung transplant: A survey of United States' transplant centers. Clin Transplant. 2019;33(7):e13630.
10. Poissy J, Damonti L, Bignon A, Khanna N, Von Kietzell M, Boggian K, et al. Risk factors for candidemia: a prospective matched case-control study. Crit Care. 2020;24(1):109.
11. Lewis TC, Sureau K, Lesko M, Rudym D, Angel L, Chang S, et al. Efficacy and Tolerability of Isavuconazole versus Posaconazole for Fungal Prophylaxis after Lung Transplantation. Journal of Heart and Lung Transplantation.

2020;39(4 Supplement):S203-S4.

12. Pennington KM, Razonable RR, Peters S, Scott JP, Wylam M, Daly RC, et al. Why do lung transplant patients discontinue triazole prophylaxis? Transpl Infect Dis. 2019;21(3):e13067.
13. Walker AM, Bohn RL, Cali C, Cook SF, Ajene AN, Sands BE. Risk factors for colon ischemia. Am J Gastroenterol. 2004;99(7):1333-7.
14. Thyagarajan V, Norman H, Alexander KA, Napalkov P, Enger C. Risk of mortality, fatal infection, and fatal malignancy related to use of anti-tumor necrosis factor-alpha biologics by rheumatoid arthritis patients. Semin Arthritis Rheum. 2012;42(3):223-33.
15. Wallace PJ, Shah ND, Dennen T, Bleicher PA, Crown WH. Optum Labs: building a novel node in the learning health care system. Health Aff (Millwood). 2014;33(7):1187-94.
16. Pennington KM, Dykhoff HJ, Yao X, Sangaralingham LR, Shah ND, Peters SG, et al. The Impact of Antifungal Prophylaxis in Lung Transplant Recipients. Annals of the American Thoracic Society. 2021;18(3):468-76.
17. Dempsey TM, Sangaralingham LR, Yao X, Sanghavi D, Shah ND, Limper AH. Clinical Effectiveness of Antifibrotic Medications for Idiopathic Pulmonary Fibrosis. Am J Respir Crit Care Med. 2019;200(2):168-74.
18. Quan H, Sundararajan V, Halfon P, Fong A, Burnand B, Luthi JC, et al. Coding algorithms for defining comorbidities in ICD-9-CM and ICD-10 administrative data. Med Care. 2005;43(11):1130-9.
19. Arendrup MC, Sulim S, Holm A, Nielsen L, Nielsen SD, Knudsen JD, et al. Diagnostic issues, clinical characteristics, and outcomes for patients with fungemia. J Clin Microbiol. 2011;49(9):3300-8.
20. Yetmar ZA, Lahr B, Brumble L, Gea Banacloche J, Steidley DE, Kushwaha S, et al. Epidemiology, risk factors, and association of antifungal prophylaxis on early invasive fungal infection in heart transplant recipients. Transplant infectious disease : an official journal of the Transplantation Society. 2021:e13714.
21. Pennington KM, Martin MJ, Murad MH, Sanborn D, Saddoughi SA, Gerberi D, et al. Risk Factors for Early Fungal Disease in Solid Organ Transplant Recipients: A Systematic Review and Meta-analysis. Transplantation.

2024;108(4):970-84.

22. Lee JH, Lee D, Kim JK, Kim HY, Hwang SD, Lee SW, et al. Efficacy and safety according to dose of valganciclovir for cytomegalovirus (CMV) prophylaxis in transplantation: Network meta-analysis using recent data.

Journal of the American Society of Nephrology. 2018;29:648.

23. Schaenman JM, Rosso F, Austin JM, Baron EJ, Gamberg P, Miller J, et al. Trends in invasive disease due to Candida species following heart and lung transplantation. Transpl Infect Dis. 2009;11(2):112-21.

1	24.	da Graca B, Filardo G, Nicewander D. Consequences for healthcare quality and research of the exclusion of 2	records from the Death Master File. Circ Cardiovasc Qual Outcomes. 2013;6(1):124-8.

3

[Click here to access/download;Figure;Figure 1.pptx](https://www2.cloud.editorialmanager.com/pone/download.aspx?id=39214948&guid=25ee2786-3d05-46bd-943f-f5c5498e6c6a&scheme=1)

All LT Coded Patients

1/1/2005-11/30/2023 N = 2,879

Patients Having LT code during

Hospitalization N = 1,588

Patients with 1 LT Hospitalization

N = 1,529

Patients with LT Hospitalization fully in

Study Period

N = 1,410

Patients with IC

N = 151

Patients without IC

N = 1,259

Clean Demographics, Logical Death Date,

No Prior IC infection

N = 1,279

Patients with IC

N = 131

Patients without IC

N = 1,148

Enrollment 90 days Prior and 7 days Post

Index Date

N = 1,287

Figure 1.

[Click here to access/download;Figure;Figure 2.pptx](https://www2.cloud.editorialmanager.com/pone/download.aspx?id=39214981&guid=d3a8544e-83c2-4a00-ac3d-20cb6a6cdc95&scheme=1)

All LT Coded Patients

1/1/2005-11/30/2023 N = 2,879

Patients Having LT code during

Hospitalization N = 1,588

Patients with 1 LT Hospitalization

N = 1,529

Patients with LT Hospitalization fully in

Study Period

N = 1,410

Patients with IC (Cases)

N = 151

Patients without IC

(

Controls

)

N = 1,259

Run Matching Algorithm

for Exhaustive

Matching up to 1

-

10

Enrollment 90 days Prior and 7 days Post

Index Date

N = 1,117

Clean Demographics, Logical Death Date,

No Prior IC infection

N = 1,076

Final 1 to 2 Match Kept in the Study

Patients with IC (Cases)

N = 126

Patients without IC

(

Controls

)

N = 252

Figure 2.

| HR: 2.31 | 95% CI: 1.45-3.67 | *P*<0.001 |
| --- | --- | --- |

[Click here to access/download;Figure;Figure 3.pptx](https://www2.cloud.editorialmanager.com/pone/download.aspx?id=39214985&guid=566b75b4-08bc-40b4-9e3f-74fe6e573e77&scheme=1) Figure 3.

[Click here to access/download;Figure;Table 1.doc](https://www2.cloud.editorialmanager.com/pone/download.aspx?id=39215007&guid=ddbf59e3-8b2d-4b5f-8749-997163436d9b&scheme=1)

**Table 1.** Non-matched cohort characteristics.

|  | **Median (IQR) or Count (%) n=1,279** |
| --- | --- |
| Age (years) | 60.0 (52.0, 66.0) |
| Male Sex | 784 (61.3%) |
| Region  Midwest  West  South and Northeast | 340 (26.6%)  171 (13.4%)  768 (60.1%) |
| Race  White  Black  Other/ Unknown | 924 (72.2%)  124 (9.7%)  231 (18.1%) |
| Reason for Transplant  COPD/ Bronchiectasis  Cystic Fibrosis  Idiopathic Pulmonary Fibrosis  Other Interstitial Lung Disease  Other | 241 (18.8%)  110 (8.6%)  619 (48.4%)  260 (20.3%)  49 (3.8%) |
| Type of Transplant  Single  Bilateral | 307 (24.0%)  972 (76.0%) |
| Follow-up (days) | 469.0 (224.0, 969.0) |
| Died during Follow-up | 289 (22.6%) |
| Days to Death from LT | 395.0 (165.0, 991.0) |
| Antifungal Medications During LT Stay | 1,194 (93.4%) |
| Antifungal Medications Prescribed During LT Stay*  Amphotericin (liposomal or deoxycholate)  Itraconazole or Fluconazole  Voriconazole, Posaconazole, or Isavuconazonium  Nystatin  Echinocandin | 50 (3.9%)  117 (9.2%)  699 (54.7%)  317 (24.8%)  166 (13.0%) |
| Developed IC | 131 (10.2%) |
| When IC Occurred (n=131)  Transplant Hospitalization  After Transplant Hospitalization | 69 (52.7%)  62 (47.3%) |
| Type of IC Based on Diagnostic Code  Pulmonary Candidiasis  Candida Sepsis  Other | 96 (73.3%)  24 (18.3%)  19 (14.5%) |
| Time from LT to IC (days) | 32.0 (0, 192.0) |

COPD- Chronic Obstructive Pulmonary Disease, IC- Invasive Candidiasis, LT- Lung Transplant

*Some patients had more than 1 antifungal medication prescribed

[Click here to access/download;Figure;Table 2.doc](https://www2.cloud.editorialmanager.com/pone/download.aspx?id=39215555&guid=bcaae54d-294f-4523-8311-7fd51d79a918&scheme=1)

**Table 2.** Characteristics of lung transplant recipients who developed invasive candidiasis compared to those who did not.

|  | **Invasive Candidiasis**  Median (IQR) or Count (%) n=131 | **No Invasive Candidiasis**  Median (IQR) or Count (%) n=1,148 | *p*-value |
| --- | --- | --- | --- |
| Age | 60.0 (51.0, 67.0) | 60.0 (52.0, 66.0) | 0.50 |
| Male Sex | 85 (64.9%) | 699 (60.9%) | 0.37 |
| Reason for Transplant  COPD/ Bronchiectasis  Cystic Fibrosis  Idiopathic Pulmonary Fibrosis  Other Interstitial Lung Disease  Other | 23 (17.6%)  <11 (<8.4%)  60 (45.8%)  35 (26.7%)  <11 (<8.4%) | 218 (19.0%)  100 (8.7%)  559 (48.7%)  225 (19.6%)  46 (4.0%) | 0.50 |
| Type of Transplant  Single  Bilateral | 34 (26.0%)  97 (74.1%) | 273 (23.8%)  875 (76.2%) | 0.58 |
| Follow-up (days) | 517.0 (245.0, 938.0) | 468.0 (220.0, 975.0) | 0.85 |
| Prescribed Corticosteroids Prior to LT Stay | 103 (78.6%) | 881 (76.7%) | 0.63 |
| High Pre-Transplant Co-morbidity  Burden^+^ | 64 (48.9%) | 590 (51.4%) | 0.58 |
| Pre-Transplant Diabetes Mellitus | 14 (10.7%) | 217 (18.9%) | 0.02* |
| Antifungal Medications During LT Stay | 117 (89.3%) | 1,077 (93.8%) | 0.05 |
| Re-operation During LT Stay | 52 (36.7%) | 313 (27.3%) | 0.003* |
| LT Hospitalization Length of Stay  (days) | 26.0 (15.0, 68.0) | 20.0 (13.0, 38.0) | <0.0001* |
| Post-Transplant ECMO | 13 (9.9%) | 90 (7.8%) | 0.41 |
| Days of ECMO Use | 11.0 (9.0, 50.0) | 8.0 (2.0, 15.0) | 0.01* |
| Renal Replacement Therapy | <11 (<8.4%) | 45 (3.9%) | 0.23 |
| CMV Disease Following Transplant | 40 (30.5%) | 293 (25.5%) | 0.22 |

CMV- Cytomegalovirus, COPD- Chronic Obstructive Pulmonary Disease, ECMO- Extracorporeal Membrane Oxygenation, LT- Lung Transplant *Denotes statistical significance. ^+^Defined Elixhauser Comorbid Conditions score of 4 or higher.

[Click here to access/download;Figure;table 3.doc](https://www2.cloud.editorialmanager.com/pone/download.aspx?id=39215556&guid=0dc37f79-b35c-419a-adbe-1cb76a7e70eb&scheme=1)

**Table 3.** Multivariable logistic regression for risk factors for invasive candidiasis following lung transplant.

| **Observations = 1,279** | **OR** | **SE** | **Z** | ***p-value*** | **95% CI** | |
| --- | --- | --- | --- | --- | --- | --- |
| **Gender** (Control Female) |  |  |  |  |  |  |
| Male Sex | 1.15 | 0.23 | 0.69 | 0.49 | 0.78 | 1.70 |
| **Transplant Reason** |  |  |  |  |  |  |
| Cystic Fibrosis | 0.70 | 0.32 | -0.76 | 0.45 | 0.28 | 1.74 |
| Idiopathic pulmonary fibrosis | 1.01 | 0.29 | 0.03 | 0.97 | 0.58 | 1.77 |
| Other Interstitial Lung Disease | 1.79 | 0.56 | 1.89 | 0.06 | 0.98 | 3.29 |
| Other | 0.55 | 0.49 | -0.67 | 0.50 | 0.10 | 3.08 |
| **Type of Transplant** (Control Single) |  |  |  |  |  |  |
| Bilateral Transplant | 0.90 | 0.20 | -0.46 | 0.64 | 0.59 | 1.39 |
| **ECMO Days** (Control ≤ 8) |  |  |  |  |  |  |
| 9+ | 2.34 | 0.99 | 2.02 | 0.04* | 1.03 | 5.34 |
| **Elixhauser** (Control ≤ 3) |  |  |  |  |  |  |
| 4+ | 0.86 | 0.30 | -0.43 | 0.67 | 0.44 | 1.70 |
| **Baseline Risk Factors** |  |  |  |  |  |  |
| CMV disease | 1.91 | 1.46 | 0.85 | 0.40 | 0.43 | 8.51 |
| Pre-Transplant Steroid Use | 1.12 | 0.28 | 0.47 | 0.64 | 0.69 | 1.83 |
| Antifungal Prescription | 1.49 | 0.42 | 1.39 | 0.17 | 0.85 | 2.59 |
| Diabetes Mellitus | 1.77 | 0.72 | 1.42 | 0.16 | 0.80 | 3.92 |
| Renal Failure | 0.75 | 0.34 | -0.65 | 0.52 | 0.31 | 1.80 |

CI- Confidence Interval, CMV- Cytomegalovirus, OR- Odds Ratio, SE- Standard Error

[Click here to access/download;Figure;Table 4.doc](https://www2.cloud.editorialmanager.com/pone/download.aspx?id=39215569&guid=e5c80426-d0ae-48aa-bdea-9dc82b7cf3f4&scheme=1)

**Table 4.** Matched cohort of lung transplant recipients with invasive candidiasis matched to age, sex, and length of hospital stay controls (1 case to 2 controls).

|  | **Invasive Candidiasis**  Median (IQR) or Count  (%) n=126 | **No Invasive**  **Candidiasis**  Median (IQR) or Count  (%) n=252 | **Standardized Difference** after match |
| --- | --- | --- | --- |
| Age | 60.0 (52.0, 67.0) | 60.0 (52.0, 66.0) | 0.01 |
| Male Sex | 81 (64.3%) | 162 (64.3%) | 0.00 |
| Reason for Transplant  COPD/ Bronchiectasis  Cystic Fibrosis  Idiopathic Pulmonary Fibrosis  Other Interstitial Lung Disease  Other | >21 (>16.7%)  <11 (<8.7%)  61 (48.4%)  33 (26.2%)  <11 (<8.7%) | 218 (19.0%)  >33 (>13.1%)  105 (41.7%)  60 (26.8%)  <22 (8.4%) | 0.32* |
| Prescribed Corticosteroids Prior to LT Stay | 108 (85.7%) | 219 (86.9%) | 0.03 |
| Pre-Transplant Diabetes Mellitus | 37 (29.4%) | 73 (29.0%) | 0.01 |
| Antifungal Medications During LT Stay | 99 (78.6%) | 212 (84.1%) | 0.11* |
| CMV Disease Following Transplant | 39 (30.1%) | 59 (23.4%) | 0.12* |

*All imbalanced variables outside of the ± 0.10 range were controlled for in the final Cox Proportional Hazard (PH) model.

Other

[Click here to access/download](https://www2.cloud.editorialmanager.com/pone/download.aspx?id=39215001&guid=7155fe23-3145-4fe5-afdc-d95f64a44bfb&scheme=1)

[Supporting Information figure legends.docx](https://www2.cloud.editorialmanager.com/pone/download.aspx?id=39215001&guid=7155fe23-3145-4fe5-afdc-d95f64a44bfb&scheme=1)
