# Supplementary material for: Invasive candidiasis following lung transplant: An Assessment of impact utilizing a national insurance claims cohort
Source: PLoS One. 2025 Aug 21;20(8):e0330162. doi: 10.1371/journal.pone.0330162 (PMC12370120; doi:10.1371/journal.pone.0330162)
Supplement: Attachment — Submitted filename: Letter to Reviewers.docx [file pone.0330162.s003.docx]

Dear Editorial Team:

We thank the editorial team and all reviewers for their time and detailed evaluation of our manuscript, *“Invasive Candidiasis Following Lung Transplant: An Assessment of Impact Utilizing a National Insurance Claims Cohort.”* We are especially grateful for the thoughtful critiques, which have significantly strengthened the clarity, methodological transparency, and overall quality of the manuscript. In the pages that follow, we provide detailed responses to each reviewer comment. All revisions made to the manuscript are noted with corresponding page and line numbers. We believe these changes enhance the rigor and impact of our work and sincerely appreciate the opportunity to revise and resubmit.

**Reviewer #1:**

We thank Reviewer #1 for their thoughtful review and valuable feedback. We are pleased that you found the topic of invasive candidiasis following lung transplantation to be of interest. Your comments helped us clarify key points and improve the overall quality of the manuscript. Our response to individual comments are outlined below:

- Please don’t use abbreviates in the abstract (ECMO).

*Thank you. This has been written in long form with abbreviation parenthetically given the commonality of the term ECMO in the healthcare profession.*

- Introduction lines 6 and 7 are wrong. Please see this article: Mayer FL, Wilson D, Hube B. Candida albicans pathogenicity mechanisms. Virulence. 2013 Feb 15;4(2):119-28. doi: 10.4161/viru.22913. Epub 2013 Jan 9. PMID: 23302789; PMCID: PMC3654610.

*Thank you for this important correction and for pointing us to the relevant literature. We agree that our original statement was inaccurate. We have revised the sentence to better reflect the known pathogenic mechanisms of Candida species, including their ability to adhere to and invade host tissues. The revised text on page 4, lines5-8 now reads:
“Candida spp. possess a range of virulence factors that facilitate epithelial adhesion, invasion, and tissue damage, contributing to pathogenesis in the setting of impaired host immunity or barrier disruption.”*

- Lines 10-11 according to reference 7 is not true.

*We thank the reviewer for this important clarification. Upon re-reviewing Reference 7, we agree that it does not support the broad statement regarding breakthrough IC with nebulized AmBisome. We have revised the sentence to remove the overstatement and better reflect the cited literature. The revised text on page 4, lines 9-11 now reads:*

*“While many lung transplant centers in the United States employ antifungal prophylaxis targeting mold infections(10), variation exists in the agents used and the duration of prophylaxis. Some studies have suggested that the use of inhaled amphotericin B without systemic antifungal prophylaxis may be insufficient to prevent all forms of invasive fungal infection, including invasive candidiasis (7).”*

- Please define invasive candidiasis “In our population, IC was defined as any diagnostic code for IC (ICD-9: 112.4, 112.5, 112.8, 112.4, 112.83, 112.5;5 ICD-10: B37.1, B37.5, B37.6, B37.7) in any position following the LT procedure”

*We appreciate the reviewer’s request for clarification. In our study, invasive candidiasis (IC) was defined using a set of International Classification of Diseases (ICD-9 and ICD-10) diagnostic codes intended to capture clinically significant invasive infections. We have added the following to Page 5, Lnes 6-9: “These codes include candidemia (e.g., 112.5, B37.7), disseminated candidiasis (e.g., 112.5, 112.83), and organ-specific invasive infections (e.g., B37.5 for candidal peritonitis and B37.6 for candidal endocarditis). We acknowledge that claims data may not distinguish between confirmed invasive disease and coding inaccuracies.”*

**Reviewer #2:**

The paper presented is a very interesting retrospective analysis of the risk factors leading to Candidiasis infection after lung transplant. The following issues regarding the statistical considerations should be addressed prior to publication:
*We thank Reviewer #2 for their thoughtful and constructive feedback. We are pleased that you	found the study of interest. We appreciate your detailed review of the statistical methodology	and have addressed each of your points below to enhance the clarity and rigor of our analysis.*

1) There appears to be selection bias in that patients with a prior history of IC were excluded, but patients with a prior exposure to anti-fungals were not. Furthermore, based on Figure 1 it appears 13% of IC cases but only 8% of non-IC cases were excluded. This suggests possible selection bias.

*We appreciate the reviewer’s observation regarding potential selection bias. Patients with a prior diagnosis of invasive candidiasis (IC) were excluded to ensure that all identified IC events occurred de novo after lung transplantation, allowing for more accurate assessment of post-transplant risk factors and outcomes. In contrast, prior antifungal exposure was not used as an exclusion criterion, as antifungals may be prescribed for a variety of prophylactic or therapeutic indications unrelated to IC and do not necessarily indicate prior infection.*

*With respect to the exclusion discrepancy noted in Figure 1, we thank the reviewer for highlighting this detail. The slightly higher exclusion rate among patients who later developed IC (13% vs. 8%) likely reflects the fact that patients with IC may have had more complex clinical courses or prolonged pre-transplant hospitalizations, during which time IC was diagnosed and coded. While this introduces a possibility of selection bias, we have added a statement acknowledging this as a limitation on page 8, line 8-11: “We acknowledge the potential for selection bias introduced by excluding patients with a history of invasive candidiasis prior to transplant while retaining those with prior antifungal exposure, which may reflect underlying differences in baseline health status. Additionally, a slightly higher exclusion rate among patients who later developed IC (13% vs. 8%) could suggest unmeasured pre-transplant differences that we were unable to fully control for.”*

2) It is unclear what the exact selection methods were for variable choices in the multivariable regression models. Based on the language Page 5 Lines 11-28, many factors were considered. However Table 3 reports the OR for only a select set of variables. For example why was age not included in the risk analysis? Similar issue for PH regression.

*Thank you for this insightful comment. We apologize for the lack of clarity regarding our variable selection process for the multivariable models. In the logistic regression model assessing risk factors for IC, we included a priori selected clinical variables based on prior literature and clinical plausibility. These included transplant-related factors (e.g., ECMO use, re-operation), demographic characteristics (e.g., sex, region), and comorbidities. Although age was considered, it was ultimately excluded from the final model due to lack of significant univariable association and collinearity with other factors such as comorbidity burden. We have now clarified this in the Methods section on page 6, lines 3-5: “Variables were selected a priori based on clinical relevance and literature review. Age was evaluated but excluded from the final model due to collinearity with comorbidity burden and lack of a significant univariable association with IC.”*

*For the Cox proportional hazards model, we used matched cohorts (on age ±5 years, sex, index date relative to transplant, and hospital length of stay), and the final model included only variables that remained imbalanced after matching. Since age was a matching variable and showed good covariate balance (standardized difference <0.1), it was not included in the final PH model. We have added text to clarify this approach in the Methods on page 6, lines 11-12: “Age was used as a matching variable (±5 years) and demonstrated good covariate balance post-matching; therefore, it was not included in the final Cox proportional hazards model.”*

3) For the PH model of mortality, it is unclear why the analysis was complicated so with the challenge of the index date for cases and controls. Why not use the index date as LT for both cases and controls, but use date of IC as a time-dependent covariate? The choice to manipulate the index date for controls opens up potential bias in the results. The overall statistical question is asking does an IC infection post LT impact overall mortality. Give that the median time from LT admission to IC was 32d (IQR 0 to 192) suggests possible confounding of analysis and interpretation of the results.

*We thank the reviewer for this important comment and agree that modeling IC as a time-dependent covariate is a valid alternative approach. Our goal, however, was to assess mortality following an episode of IC, and to compare outcomes in those with and without IC who were at a similar timepoint post-transplant and had similar post-operative trajectories (as reflected by hospital length of stay and index date proximity). To do this, we aligned the timing of follow-up between cases and controls by anchoring the index date for each control to mirror the case's time from transplant to IC diagnosis, thus mitigating immortal time bias and facilitating interpretation of post-IC outcomes.*

*We recognize that this approach may introduce its own limitations, particularly with regard to assumptions around matching and event timing. We have acknowledged this as a limitation in the Discussion page 8, lines 34-38: “Our analytic approach to evaluating post-IC mortality involved assigning matched controls a pseudo-index date aligned with the timing of IC diagnosis in cases. While this allowed for comparison of outcomes from similar post-transplant timepoints, it may introduce bias if the assumptions underlying index date alignment are not fully met. Modeling IC as a time-dependent covariate in a Cox model represents an alternative approach and may help validate our findings in future studies.”*

4) Page 6, Lines 49-50: As over 90% of subjects had antifungal prophylactic therapy it is not surprising there is no observed benefit.

*We agree with the reviewer that the high prevalence of antifungal prophylaxis in our cohort likely limited our ability to detect a protective association. This high background rate reduces variability in exposure, potentially obscuring any true effect of prophylaxis on invasive candidiasis (IC) risk. We have added language to the Discussion on page 7, lines 44-48: “Given that over 90% of patients received antifungal prophylaxis, our ability to detect a protective effect was likely limited by the lack of variability in exposure, and the absence of observed benefit should be interpreted with caution.”*

5) Page 6, Lines 47-54: this paragraph is a reiteration of the data in Table 3.

*We appreciate the reviewer’s perspective. While the content in this paragraph does overlap with	Table 3, we believe that summarizing key findings from the multivariable analysis in the text	improves accessibility and supports interpretation for readers who may not focus on tables	alone. These results help contextualize the lack of association for several anticipated risk factors,	such as CMV disease and diabetes mellitus, and emphasize that only prolonged ECMO support	was statistically significant. We have reviewed the paragraph to ensure it remains concise and	non-redundant and believe its inclusion strengthens the narrative presentation of our findings.	Edited text is on page 6, lines 54-60: “Post-transplant ECMO (OR: 2.34; 95% CI 1.03 to 5.34, p	=0.043) use of greater than 8 days was the only risk factor significantly associated with post-	transplant IC on multivariable modeling. Other clinical factors, including the presence of	antifungal prophylaxis during the 90 days prior to IC (OR: 1.49; 95% CI 0.85 to 2.59, p=0.165),	CMV disease (OR: 1.91; 95% CI 0.43 to 8.51, p = 0.395), diabetes mellitus (OR: 1.77; 95% CI	0.80 to 3.92, p=0.156), high co-morbidity burden (OR: 0.86; 95% CI 0.44 to 1.70, p=0.67),	bilateral lung transplant (OR: 0.90; 95% CI 0.59 to 1.39, p=0.643), and pre-transplant steroid	use (OR: 1.12; 95% CI 0.69, 1.83, p=0.641) were not significant risk factors for IC. No single	indication for transplant increased the odds of IC.*

6) Why are the results of the PH model not shown as a table? The HR in Figure 3 should be that from the PH model, and the legend should clarify this.

*We appreciate the reviewer’s suggestion to enhance clarity. We have added a new table (now	Table 5) summarizing the results of the Cox proportional hazards model, including the hazard	ratio, 95% confidence interval, and p-value. This allows for clearer interpretation and	complements the Kaplan-Meier curve presented in Figure 3. We have also updated the Figure 3	legend to specify that the hazard ratio shown is derived from the Cox model.*

*Figure 3 Legend now states, “Kaplan-Meier survival curve comparing lung transplant recipients	with and without invasive candidiasis (IC). The hazard ratio (HR: 2.31; 95% CI: 1.45–3.67) is	derived from the Cox proportional hazards model adjusted for variables with residual imbalance	following matching.”*

*Results section on page 7, lines 7-10 now states, “****Table 5*** *presents the results of the Cox	proportional hazards model evaluating the impact of IC on all-cause mortality. All-cause	mortality was significantly higher in those who developed IC (event rate per 100 person-years:	11.32; HR: 2.13; 95% CI 1.45 to 3.12, p<0.001). This held true when matching IC cases to	controls (event rate per 100 person-years: 12.87; HR: 2.31; 95% CI 1.45 to 3.67, p<0.001)	(****Figure 3****).*

Minor issues:
1) Abstract indicates data was collected January 1, 2005, to July 31, 2023 while Page 4 Lines 49-50 indicate data study period was January 1, 2005 to December 31, 2023. Suggest abstract should be revised.

*Thank you for identifying this inconsistency. We have revised the abstract to reflect the correct	study period of January 1, 2005, to December 31, 2023, in alignment with the Methods section.*

2) Revise Page 5 Line 43 to read more clearly: “Analysis of both risk factors in the development of IC and mortality from IC required us to use two different models and extraction methods.”

Thank you. We have updated this statementon on page 5, line 47-48.

3) Table 1 and Table 2 should be combined with the first column reporting cohort total, second column IC, third column non-IC.

*We appreciate the reviewer’s suggestion. We considered combining Tables 1 and 2 but felt that	presenting baseline characteristics (Table 1) and post-transplant outcomes (Table 2) separately	improves readability and keeps the focus clear for each table. Combining them would result in a	substantially larger and denser table, potentially making it more difficult for readers to interpret	key findings. We have retained the current format to preserve clarity and emphasize the	distinction between baseline variables and outcome data.*

4) Table 3 can exclude the Z-score value. Also “Control” is an improper term, use “Reference” instead.

*Thank you for this helpful suggestion. We have removed the Z-score column from Table 3 and	replaced the term “Control” with “Reference” to more appropriately indicate the referent	category in the regression model.*

**Reviewer #3:**

*We thank Reviewer #3 for their thoughtful review and constructive feedback. We appreciate your time and expertise in evaluating our manuscript and have carefully addressed each of your comments to improve the clarity, accuracy, and overall quality of the work.*

(abstract) Write the aim of the study at past tense.

*This has been updated on page 3, lines 3-4: “This study aimed to assess the incidence, risk	factors, and impact of IC on mortality in LTRs using a national insurance claims cohort.”*

(abstract) Define ECMO abbreviation.

*This has been done at the suggestion of Reviewer 1.*

(general comment) Do not start a sentence with an abbreviation.

*Thank you for this editorial suggestion. We have reviewed the manuscript and revised sentences	that began with abbreviations to ensure proper formatting and improved readability throughout	the text.*

(abstract)Define "index hospitalization".

*We have updated the abstract on page 3, lines 9-11: “IC occurs in approximately 10% of LTRs,	primarily during the initial hospitalization for lung transplantation (index hospitalization).”*

*We also updated the methods on page 5, lines 26-29: “Post-transplant re-operation was defined	as a procedure code with description of “chest closure”, “chest exploration” or “chest washout”	(CPT: 00520, 21750, 35820; ICD-9: 3451, 3452) occurring more than 24 hours after the initial	transplant procedure and within the initial hospitalization for lung transplantation (index	hospitalization).”*

(introduction) Please be specific when you say "at high risk".

*Thank you for this suggestion. We have revised the sentence in the Introduction to provide	greater specificity by referencing the incidence of invasive fungal infections (IFIs) and associated	mortality risk in lung transplant recipients, as reported in prior studies.*

*Page 4, lines 2-4 now read, “Lung transplant recipients (LTRs) are particularly vulnerable to	invasive fungal infections (IFIs), which occur in up to 25% of recipients and are linked to an	alarming three-fold increase in all-cause mortality(1-3).”*

(introduction) Define early in "early post-lung transplant period"

*Thank you for this helpful suggestion. We have revised the text to clarify that “early post-lung	transplant period” refers to the first 90 days following transplantation, during which the majority	of IC cases occur.*

*Page 4, lines 3-6: “While Aspergillus spp. have traditionally been seen as the primary cause of IFI	in LTRs(4-6), more recent single-center studies suggest that invasive candidiasis (IC) may also be	an important cause of IFI, particularly within the first 90 days post-lung transplant period(7, 8).”*

(introduction) Please provide the frequencies for "antifungal medications can have significant adverse effects".

*Thank you for this suggestion. We have revised the sentence to include representative	frequencies of adverse effects associated with antifungal medications, based on published	literature.*

*Page 4, lines 21-24: Moreover, antifungal medications can have significant adverse effects, with	reported rates including QTc prolongation in up to 20% of patients, hepatotoxicity in 5–15%,	periostitis in approximately 10% of those receiving long-term voriconazole, and increased risk of	skin cancer with prolonged triazole exposure(12, 13).*

(introduction) Write the aim of the study at past tense.

*Thank you. This has been updated.*

(methods) Who can access OLDW? It is open access?

*We appreciate the request for clarification. The OptumLabs Data Warehouse (OLDW) is not open	access. Access is granted through an annual call for proposals, where selected researchers	receive funded research credits. Additionally, researchers with external funding may access	OLDW data by adhering to the fee schedule outlined on the UC microsite.*

(methods) "Re-transplantations were excluded" why?

*Thank you for this important question. We excluded re-transplantation cases to minimize	heterogeneity in the study population. Patients undergoing re-transplantation may have distinct	baseline characteristics, higher comorbidity burden, and different risk profiles for invasive	candidiasis compared to primary lung transplant recipients. Including these patients could	introduce confounding and limit the generalizability of our findings. We have added a brief	rationale to the Methods section for clarity.*

*Page 5, lines 2-4: “Re-transplantation cases were excluded to reduce heterogeneity, as these	patients often have distinct clinical characteristics and risk profiles compared to primary	transplant recipients.”*

(methods) Detail the method use to collect mortality data.

*Thank you for this comment. We have expanded the Methods section to clarify how mortality	data were collected. Mortality was primarily ascertained using the Social Security Death Master	File, which was supplemented with hospital discharge status and insurance disenrollment data	flagged as death-related. This multi-source approach was used to enhance completeness,	particularly for inpatient deaths. We have now clarified this in the Methods section.*

*Page 5, lines 17-20: “Mortality data were primarily obtained from the Social Security Death	Master File, a national database that tracks death records. To increase completeness, particularly	for deaths occurring during hospitalization, this was supplemented with information from	hospital discharge status codes and insurance disenrollment records explicitly attributed to	death. This approach has been validated in prior studies using the OptumLabs Data	Warehouse(17, 18).”*

(methods) Detail the Elixhauser comorbidity score.

*Thank you for this helpful comment. We have revised the Methods section to provide additional	detail on how the Elixhauser comorbidity score was calculated. The score was derived using ICD-	9 and ICD-10 diagnostic codes from claims data during the 90-day baseline period prior to	transplant, applying the Quan et al. algorithm. This method is widely used for claims-based	comorbidity adjustment and has been validated in multiple populations.*

*Page 5, lines 23-25: “Elixhauser comorbidity score was calculated using ICD-9 and ICD-10	diagnostic codes captured during the 90-day baseline period prior to transplant, applying the	Quan et al. adaptation of the Elixhauser algorithm(19).”*

(methods) "compared using a t test" even those with deviations from the theoretical normal distribution?

*We appreciate the reviewer’s attention to statistical rigor. We agree that the t test assumes	approximate normality of the underlying distribution. For continuous variables, we assessed	distributional characteristics and used either the Student’s t test or the Wilcoxon rank-sum test	as appropriate. We have revised the Methods section to reflect that non-parametric tests were	used when normality assumptions were not met.*

*Page 5, lines 51-55: “Continuous variables were expressed as mean ± standard deviation (SD) or	median with interquartile range (IQR), as appropriate and were compared using Student’s t test	or the Wilcoxon rank-sum test, depending on distributional characteristics assessed by visual	inspection and normality testing.”*

(methods) Describe the methods used to decide which variables entered in multivariable model.

*Thank you. We have clarified this on page 6, lines 12-17: “A multivariable logistic regression	model was used to investigate which risk factors were associated with IC. Variables were selected	a priori based on clinical relevance and literature review. Age was evaluated but excluded from	the final model due to collinearity with comorbidity burden and lack of a significant univariable	association with IC.”*

(methods) "we matched one case" the case is defined as patient with IF? Please clarify.

*Thank you for this request for clarification. In our study, the term "case" refers specifically to	lung transplant recipients who developed invasive candidiasis (IC) following transplant.*

*Page 6, lines 20-23: “For this analysis, we matched one case—defined as a LTR who developed	IC—to two controls on age ±5 years, sex, index date with respect to the LT admission date, and	length of hospital stay.”*

(methods) Tell the readers the level of significance used in the statistical analysis.

Thank you for pointing this out. We have added a statement to the Methods section indicating	that a two-sided p-value of <0.05 was considered statistically significant for all analyses.

*Page 6, line 39: “All statistical tests were two-sided, and p-values were reported to quantify the	strength of evidence against the null hypothesis. A p-value threshold of <0.05 was used as a	general reference for interpreting the results.”*

(results) Define axes and units of measurements on Kaplan-Meier graph.

*Thank you for this comment. We have revised the figure legend and axis labels to clarify that the	x-axis represents time since index date in years, and the y-axis represents the cumulative	probability of survival. The event of interest is death.*

*Figure 3 legend: “Kaplan-Meier survival curve comparing lung transplant recipients with and	without invasive candidiasis (IC). The x-axis represents time from index date (in years), and the y-	axis represents the probability of survival. The hazard ratio (HR: 2.31; 95% CI: 1.45–3.67) is	derived from the Cox proportional hazards model, adjusted for residual covariate imbalance	after matching. The event of interest is all-cause mortality.”*

(results) Table 1 - 4: do not include the units of measurements in the body of the table. Generally, when round brackets are used the lower and upper values are not included in the range; please clarify.

*Thank you for this formatting recommendation. We have revised Tables 1 through 4 to remove	units of measurement from the body of the tables. Units are now reported in the table footnotes	or column headers where appropriate. We also clarified the use of parentheses for ranges and	interquartile ranges (IQR), and have noted explicitly that the values are inclusive. This improves	consistency and aligns with standard formatting conventions.*

(results) Report p-values with at least 3 decimals

*Thank you for this suggestion. We have updated the presentation of p-values in the tables and	text to include at least three decimal places to align with common reporting standards and	enhance precision, particularly for values near conventional significance thresholds.*

(discussion) "Our study utilized administrative claims data to evaluate both risk factors and the impact of IC in a diverse cohort of LTRs." This is duplicated information and should be deleted.

*Thank you for pointing out the redundancy. We have removed the duplicated sentence from the	Discussion to improve clarity and avoid repetition.*

(discussion) Are your results expected to be confirm on other cohorts?

*We appreciate the reviewer’s question. While our findings are consistent with prior single-center	studies, confirmation in other cohorts—particularly those with different patient populations,	prophylaxis strategies, or healthcare systems—is warranted. We have added a statement to the	Discussion to acknowledge the need for external validation.*

*Page 8, lines 14-17: “While our findings align with prior single-center studies, confirmation in	other cohorts—particularly those with different antifungal prophylaxis practices or transplant	protocols—will be important to validate these results and assess generalizability.”*

(discussion) Discuss the practical utility of the reported results.

*Thank you for this important suggestion. We have added a paragraph to the Discussion	highlighting the practical implications of our findings, particularly for risk stratification,	antifungal stewardship, and early post-transplant management strategies.*

*Page 8, line15-22: “Our findings do have practical implications for clinical management and	resource allocation in LT care. Identifying prolonged ECMO support as a significant risk factor	for IC highlights a high-risk subgroup where heightened surveillance or targeted antifungal	strategies may be warranted. Additionally, the observed association between IC and increased	mortality underscores the need for early recognition and timely intervention. The data also	inform	antifungal stewardship efforts by emphasizing that universal prophylaxis may not fully	prevent IC and should be balanced against drug-related toxicities. As such, these results support	a risk-based approach to IC prevention and may help guide clinical decision-making in the early	post-transplant period.”*

**Reviewer #4:**

*We thank Reviewer #4 for their thoughtful review and valuable feedback. We appreciate your careful evaluation of our manuscript and have addressed each of your comments below to strengthen the clarity, scientific rigor, and overall impact of the work.*

This article does not mention diagnostic methods. How is pulmonary candidiasis diagnosed? The medications taken by the patient were not mentioned in this article. How much did their use contribute to the disease's development? What is the diagnostic code for IC?

*We thank the reviewer for these questions and appreciate the opportunity to clarify. As noted in	the Methods section, our study utilized administrative claims data from the OptumLabs® Data	Warehouse. Diagnostic methods, such as microbiologic or radiologic findings, are not available	in claims-based datasets. Instead, cases of invasive candidiasis (IC) were identified using specific	ICD-9 and ICD-10 diagnosis codes (ICD-9: 112.4, 112.5, 112.8, 112.83; ICD-10: B37.1, B37.5,	B37.6, B37.7), as outlined in the Methods section.*

*Regarding medications, we examined antifungal prescriptions within 90 days prior to the IC	diagnosis and reported their frequency in the Results. These were included in our multivariable	analysis to assess their association with IC development. However, as this is a claims-based	study, we did not have access to medication indication, duration, or microbiologic data to	determine causality.*

**Reviewer #5:**

Congratulations to this mostly very well written manuscript. It has been one of very few manuscripts that I reviewed during the last few years that didn't have any major statistical flaws. The only reason that I selected major instead of minor revision is the number of questions that I have and that should be clarified before this manuscript is published. I hope they are helpful:

*We sincerely thank Reviewer #5 for their thoughtful and generous feedback. We are especially grateful for your positive comments regarding the clarity of the manuscript and the statistical rigor of the analysis. We have carefully addressed each of your questions and suggestions below to further strengthen the manuscript and ensure clarity for readers.*

1. The authors write in the Statistical Analysis section that in the descriptive tables, they show mean + sd or median and IQR as appropriate. However, in Tables 1 and 2 there only is the median/IQR shown. Is the reason for this that all variables seem to be nonnormally distributed? Or just for was of presentation (to avoid confusions due to switching between mean and median)? In any case, only the median/IQR should be mentioned in the methods.

*We thank the reviewer for this insightful observation. We chose to present all continuous	variables using median and interquartile range (IQR) for consistency and ease of interpretation,	particularly given the skewed distributions of several variables. To avoid confusion, we have	revised the Methods section to state that continuous variables are presented as median (IQR)	only.*

2. Follow-up question from the above: As far as I understood, all tests performed in Table 2 are t-tests? But if the (or some of the) data are nonnormally distributed, a nonparametric test should be used. Please change that.

*Thank you for this important clarification. We agree that nonparametric testing is more	appropriate for non-normally distributed data. As noted in our revised response to Reviewer #3,	we used the Wilcoxon rank-sum test for continuous variables that were not normally distributed.	We have clarified this in the Methods section and confirmed that the appropriate test was	applied to each variable in Table 2.*

*Page 5, lines 51-55: Continuous variables were expressed as median with interquartile range	(IQR), as appropriate and were compared using Student’s t test or the Wilcoxon rank-sum test,	depending on distributional characteristics assessed by visual inspection and normality testing.	The tables were analyzed across the case and control groups to test for differences.*

3. Please delete the stars denoting statistical significance from the tables. Nowadays, there is a strong trend towards quantifying the evidence against the null hypothesis instead of making a binary cutoff to distinguish "significant" from "non-significant". I don't mind you discussing significance in the text (although I clearly prefer writing something like "There was no evidence for a difference"), but do not use stars in the tables.

*We appreciate this excellent point and fully agree with the shift toward emphasizing effect	estimates and p-values rather than dichotomous interpretations. We have removed the asterisks	denoting statistical significance from all tables to better reflect this approach. We also removed	the term significance from the paper to allow focus on strength of relationships.*

4. How were the covariates for the multivariable logistic regression model chosen? I don't fully understand the line "We then assessed a priori which risk factors were associated." How did you do that? Is that a fancy way of writing "We asked experts which risk factors to include"? Please clarify.
By the way, I very much like that you obviously didn't choose the covariates based on their p-value or something wrong like that. But some information how you actually chose them would be very helpful.

*We greatly appreciate the reviewer’s positive comments regarding our covariate selection	approach and agree that additional clarification was warranted. We have revised the Methods	section to specify that variables were selected a priori based on clinical relevance and review of	prior literature (we did a SR/MA on risk factors in SOT published in Transplantation	DOI:* [*10.1097/TP.0000000000004871*](https://doi.org/10.1097/tp.0000000000004871)*), rather than based on statistical significance or	univariable screening. While subject-matter expertise informed our choices, the process was	structured around previously published studies and biologic plausibility. We also added	clarification on the exclusion of age due to collinearity and lack of univariable association.*

*The revised text on page 6, lines 10-14: "A multivariable logistic regression model was used to	investigate which risk factors were associated with IC. Variables were selected a priori based on	clinical relevance and literature review. Age was evaluated but excluded from the final model due	to collinearity with comorbidity burden and lack of a univariable association with IC..."*

5. I recommend to use the word "Reference category" instead of "Control" in Table 3. Control can be misunderstood as being a control group, especially as you also have matching in your manuscript.

*Thank you. This has been updated.*

6. Both the ECMO days and the Elixhauser variable were dichotomized, but for example in Table 2 the Elixhauser is included as count variable. I don't like dichotomizing very much because you usually loose information. Have you tried to include them in a continuous way? If so, what was the result? Have you looked at the descriptives if it might be reasonable to include them in a non-linear way? And if that is not an option (with a good reason): How were the thresholds chosen? If there is a clinical reasoning behind that (if for example the threshold is used in clinical practice) this should be stated and a reference for this should be given. If the threshold was selected by the authors, please also write how and why you did that.

*We thank the reviewer for this excellent comment and completely agree that dichotomization	can result in loss of information. We originally dichotomized the Elixhauser comorbidity index	and ECMO days to simplify interpretation and enhance clinical relevance. For the Elixhauser	score, we used a cutoff of >4 to reflect a high comorbidity burden based on distributional	characteristics in our dataset and consistency with thresholds used in prior studies involving	transplant or critically ill populations (e.g., Quan et al., 2005). We have added a reference and	clarified this in the Methods.*

*For ECMO, we dichotomized at >8 days based on observed distribution in our cohort and prior	literature suggesting that extended duration beyond one week may increase risk of	complications. However, we acknowledge that modeling ECMO duration and comorbidity	burden as continuous or non-linear terms could yield additional insights. We have added a	statement to the Discussion noting this limitation and suggesting it as an area for future study.*

*Page 5, lines 22-29: “Independent variables of interest included sex, age, race/ethnicity, census	region, single versus double lung transplant, and indication for transplant.* *Elixhauser	comorbidity score was calculated using ICD-9 and ICD-10 diagnostic codes captured during the	90-day baseline period prior to transplant, applying the Quan et al. adaptation of the Elixhauser	algorithm(19). The Elixhauser comorbidity index was dichotomized at ≥4 to define high	comorbidity burden, consistent with distributional patterns in our data and previous literature.	ECMO duration was dichotomized at >8 days based on prior studies linking prolonged (>7 days)	ECMO support to increased post-transplant complications(20).”*

*Page 8, lines 59-61: “We dichotomized ECMO duration and comorbidity burden for ease of	interpretation and consistency with prior studies, but acknowledge this approach may have	limited our ability to detect dose-response relationships.”*

7. Two hazard ratios are mentioned in the text. First, 2.13, then 2.31 "when matching IC cases to controls", and the latter one is also printed below the Kaplan-Meier curve. I don't understand which model the first value comes from. Is that a simple, unmatched model with just one influential variable? I also find it irritating that the value 2.31 comes from the adjusted model that the authors mention, but that it is printed below the KM curve suggests to me that it comes from an unadjusted model. Please clarify. Think about not showing the HR below the curve, as the HR comes from an adjusted model.

*We appreciate the reviewer’s close attention to this important point. To clarify, the hazard ratio	of 2.13 reflects the unadjusted association between invasive candidiasis (IC) and mortality in the	full cohort prior to matching, while the hazard ratio of 2.31 was derived from the adjusted Cox	proportional hazards model applied to the matched cohort*.

*We agree that displaying the adjusted HR below the Kaplan-Meier curve may lead to confusion,	as the KM curve is an unadjusted visual representation. In response to this feedback, we have	removed the HR from the figure and clarified in the* *figure legend that the HR of	2.31 originates	from the adjusted model used in the matched analysis.*

*Figure 3 legend: “Kaplan-Meier survival curve comparing lung transplant recipients with and	without invasive candidiasis (IC). The x-axis represents time from index date (in years), and the	y-axis represents the probability of survival. The curves reflect unadjusted survival estimates. The	effect of IC on all-cause mortality was further assessed using a Cox proportional hazards model,	adjusted for residual covariate imbalance following matching (adjusted HR: 2.31; 95% CI: 1.45–	3.67), as reported in the Results section.*

8. Please print the results of the final Cox model in an additional table. I think it important to also see what hazard ratios the other influential variables show.
*We appreciate the reviewer’s suggestion to enhance clarity. We have added a new table (now	Table 5) summarizing the results of the Cox proportional hazards model, including the hazard	ratio, 95% confidence interval, and p-value.*

9. Did you check the proportional hazards assumption? Please mention that and the results of this.

*Thank you for this important methodological point. We evaluated the proportional hazards	assumption for the Cox model using Schoenfeld residuals. The assumption was not violated, and	the model was deemed appropriate for	estimating hazard ratios over time. We have added a	statement to the Methods section to reflect this assessment.*

*Page 6, lines 28-29: The proportional hazards assumption was assessed using Schoenfeld	residuals, and no violations were identified.*

10. I don't understand the > and < signs in Table 4. Please explain.

*Thank you for pointing this out. The “<” and “>” symbols in Table 4 reflect data suppression	required by OptumLabs® Data Warehouse privacy policies, which prohibit reporting exact counts	when cell sizes are ≤11 to protect patient confidentiality. We have clarified this in the table	footnote for transparency.*

*Footnote: “Per OptumLabs® data use policies, cell counts ≤11 are masked and reported using	“<” or “>” symbols to protect patient privacy.”*

**Reviewer #6:**

I read with great interest the article entitled “Invasive Candidiasis Following Lung Transplant: An Assessment of Impact Utilizing a National Insurance Claims Cohort”, which falls within the aim of this journal. This study aimed to assess the incidence, risk factors, and impact of invasive candidiasis on mortality in lung transplant recipients using administrative claims data from individuals enrolled in commercial and Medicare Advantage health plans in the US.
In my honest opinion, the topic and results are interesting for audiences and the paper is almost well structured enough to attract the readers’ attention. However, the authors should consider and clarify some points and improve the paper, as suggested below [*: major points, #: minor points].

*We thank Reviewer #6 for their thoughtful review and kind comments regarding the structure, relevance, and potential impact of our manuscript. We appreciate your constructive suggestions and have carefully addressed each of your points below to further improve the clarity and quality of the paper.*

Abstract section
\# Please note that the tone of your study’s objective in the Introduction part should align with the verb tense used in the Methods and Results parts. Therefore, instead of “This study aims to…”, consider using ‘This study aimed to….’.

*Thank you. We have made this modification as per a prior reviewer also.*

\# Please ensure that the final sentence of the Results part clarifies whether the finding is statistically significant. Therefore, include the relevant statistical measures.

Thank you for this helpful suggestion. In alignment with other reviewer feedback, we have	avoided the use of “statistically significant” language, but have revised the sentence to include	the appropriate statistical measures to allow readers to interpret the results based on effect size	and confidence intervals.

Page 3, lines 14-16: “Mortality was higher in LTRs with IC (HR: 2.31; 95% CI: 1.45 to 3.67; p <	0.001). LTRs with IC also had longer hospital stays (median 26.0 days vs. 20.0 days; p < 0.001)	and more re-operations (36.7% vs. 27.3%; p = 0.003) compared to those without IC.”

* Writing the Conclusion part is crucial, but it is currently insufficient. Additionally, the first sentence is very similar to the first line of the previous part. So please revise it. You may use the following suggestion or a similar one: ‘Invasive candidiasis affects approximately 10% of lung transplant recipients and is linked to higher mortality, prolonged hospitalization, and increased surgical interventions. These findings underscore the importance of early identification and targeted preventive strategies to improve post-transplant outcomes’.

*Thank you for this helpful feedback. We agree that the original conclusion was repetitive and	insufficiently emphasized the clinical impact of our findings. We have revised the conclusion to	improve clarity, eliminate redundancy, and highlight the importance of prevention and early	recognition.*

*Page 3, lines 18-21: “Invasive candidiasis affects approximately 10% of lung transplant recipients,	most often during the initial hospitalization. It is associated with increased mortality, prolonged	hospital stays, and a greater need for surgical re-intervention. These findings highlight the	importance of early identification and targeted preventive strategies to improve outcomes in this	high-risk population.”*

Introduction section
* Please note that the phrase “Since Candida spp. do not have the ability to digest and invade tissues” is not entirely accurate based on various studies, including this one: <https://doi.org/10.1016/j.rsma.2023.103258>. So kindly revise it.

Thank you for this important correction. We agree that the original phrasing did not reflect	current understanding of Candida spp. pathogenicity. Based on this and prior reviewer feedback,	we have revised the sentence to state:

Page 4, lines 6-9: *“Candida spp. possess virulence factors that promote adhesion to host tissues,	hyphal transformation, and enzymatic degradation of epithelial barriers, allowing for tissue	invasion and dissemination, particularly in immunocompromised hosts.”*

\# Please note that the phrase “its effectiveness has not been established in the lung transplant population” is stated too definitively. It would be preferable to rephrase it as ‘evidence exists but is inconclusive’.

*Thank you for this helpful suggestion. We agree that the original phrasing was too definitive. We	have revised the sentence to more accurately reflect the current state of the evidence, which is	limited and inconclusive.*

*Page 4, lines 20-24: “While universal antifungal prophylaxis may seem like an effective	preventative measure for IC, evidence in the lung transplant population remains limited and	inconclusive.”*

\# Please note that here “One tool that has been utilized to assess clinical incidence and risk factor questions in other clinical domains are large claims databases, such as OptumLabs® Data Warehouse”, “One tool” does not align with “are”. It is recommended to restructure the sentence. A suggested revision is: ‘Large claims databases, such as OptumLabs® Data Warehouse (OLDW), have been widely used to assess disease incidence and risk factors across various clinical domains’.

*Thank you for identifying this grammatical issue and for providing a clear and well-phrased	revision. We have updated the sentence in the manuscript to reflect your suggestion.*

*Page 4, lines 30-32: “Large claims databases, such as OptumLabs® Data Warehouse (OLDW),	have been widely used to assess disease incidence and risk factors across various clinical	domains(14, 15).”*

Methods section
* Regarding your sentence: “We required subjects to have at least 90 days of continuous health plan coverage prior to their lung transplant date and at least 30 days post”. Please clarify the rationale behind selecting the 90-day pre-transplant and 30-day post-transplant timeframes.

*Thank you for this helpful question. The 90-day pre-transplant enrollment period was selected to	ensure adequate baseline data for assessing comorbidities and medication use. This duration is	commonly used in claims-based studies to capture relevant clinical history prior to an index	event. The 30-day post-transplant enrollment minimum was applied to ensure that patients had	sufficient follow-up for identification of early post-transplant outcomes, including invasive	candidiasis. We have added this rationale to the Methods section for clarity.*

*Page 4, lines 57-60: “We required at least 90 days of continuous enrollment prior to transplant to	allow for adequate baseline characterization, and at least 30 days post-transplant to ensure	sufficient follow-up to assess early post-transplant outcomes.”*

\# In the part Outcomes of Interest, please note that ICD code 112.5 appears to be duplicated.

*Thank you. We fixed this duplication.*

* Since your study follows a cohort design, it is preferable not to use the terms “Case” and “Control”, which are more relevant to case-control studies. Please revise these terms throughout the text and figures, replacing them with ‘diseased’ and ‘non-diseased’, respectively. Additionally, when referring to mortality outcomes, please use ‘exposed’ and ‘unexposed’ instead.

*We appreciate this thoughtful clarification regarding terminology. While we used the terms	“case” and “control” to describe matched groups for mortality analysis, we agree that this	language is more appropriate for case-control designs. To reflect the cohort nature of our study,	we have revised the manuscript to use the terms “IC” and “non-IC” to refer to patients with and	without invasive candidiasis, respectively. In the context of mortality analyses, we now refer to	these groups as “exposed” (IC) and “unexposed” (non-IC). These changes have been made	throughout the text, tables, and figure legends to improve accuracy and clarity.*

\# In the Mortality Analysis part, it would be preferable to briefly explain the rationale behind matching for the selected variables. For instance for confounding variables.

*Thank you for this helpful suggestion. We have revised the Methods section to include a brief	rationale for matching. The selected variables—age, sex, transplant date proximity, and length of	hospital stay—were chosen to reduce confounding by factors known to influence both IC	development and post-transplant mortality.*

*Page 6, line 23-28: “**For this analysis, we matched one exposed—defined as a lung transplant	recipient (LTR) who developed IC—to two unexposed on age (±5 years), sex, index date with	respect to the LT admission date, and length of hospital stay. These variables were selected to	control for potential confounders known to influence both the development of IC and post-	transplant mortality. Age was used as a matching variable and demonstrated good covariate	balance post-matching; therefore, it was not included in the final Cox proportional hazards	model.*

Results section
\# Please ensure consistency in punctuation. In this instance, (619; 48.4%) uses a semicolon, whereas a comma would be more appropriate for consistency.

*Thank you. This has been corrected.*

* Please note that (Q1, Q3) is not equivalent to the interquartile range (IQR), as IQR is calculated as Q3 minus Q1. It is recommended to report IQR as a single value in the text while maintaining the (Q1, Q3) format in tables for clarity.

*Thank you for this clarification. We agree that the interquartile range (IQR) should be presented	as a single value in the text, while the (Q1, Q3) format provides clearer context in tables. We	have updated the text accordingly to report IQR as a single value and retained the (Q1, Q3)	format in all tables.*

\# Regarding the sentence “The most commonly prescribed antifungal were mold-active azoles”, please note that “antifungal” should be written as ‘antifungals’ or, preferably, ‘Antifungal Medications’ to maintain consistency with the terminology used in the tables.

*Thank you. This has been updated.*

\# In the first paragraph of this section, please remove all instances of (n, x%) as this information is already provided in the table.

*Thank you for this suggestion. We have removed the redundant (n, x%) values from the first	paragraph of the Results section to improve readability and avoid duplication of information	already presented in Table 1.*

\# Please revise the sentence “Of these, 131 (10.2%) LTRs developed IC following LT”. to explicitly refer to ‘lung transplant recipients’ instead of using “Of these”.

*Thank you for the suggestion. We have revised the sentence to improve clarity by explicitly	referring to lung transplant recipients.*

*Among the 1,279 LTRs included in the study, 131 (10.2%) developed IC following transplant.*

* Preferably, ensure that the variable titles used in the tables are consistent with those in the main text. For example, term like “locally invasive candidiasis/deep-seated candidiasis” in the text are somewhat ambiguous, whereas the table clearly refers to “Pulmonary Candidiasis”. Please make the necessary revisions and include any additional explanations in parentheses.

*Thank you. We revised the table to be consistent with the text.*

\# Please clarify what you mean by “IC diagnostic codes are limited”.

*Thank you for this important point. We agree that this phrasing was vague and have revised the	sentence for clarity. Our intent was to acknowledge that administrative claims data rely on ICD	codes, which may lack clinical granularity and do not distinguish between different anatomical	sites or severity levels of invasive candidiasis. This limitation affects our ability to fully	characterize the nature of IC cases in the cohort.*

*Page 7, lines 2-7: “Invasive candiasis diagnostic codes in administrative claims data are limited	in clinical detail and do not distinguish between specific anatomical sites (e.g., thoracic vs.	bloodstream) or severity of infection, which may affect the classification of IC presentations in	this study.”*

* In the Invasive Candidiasis part, please remove all instances of (n, x%) since this information is already provided in the tables. Additionally, mentioning other details is unnecessary; instead, simply state that all had a P-value < 0.05 (also please clarify that if you have considered this threshold in the Statistical Analysis part). Please apply this recommendation to other parts as well.

*Thank you for this helpful suggestion. We have removed (n, x%) values where this information is	already available in the tables to reduce redundancy. In keeping with guidance from other	reviewers, we have not used a binary threshold of significance but instead report exact p-values	(to three decimal places) to allow readers to interpret the strength of evidence. We have also	revised the Statistical Analysis section to reflect that p-values are reported descriptively and not	used as strict cutoff values for interpretation.*

\# For the sentence: “Post-transplant ECMO (OR: 2.34; 95% CI 1.03 to 5.34, p = 0.043) use of greater than 8 days was the only risk factor significantly associated with post-transplant IC on multivariable modeling”. Please remove the information inside the parentheses as previously suggested. Additionally, after “associated with”, add ‘was associated with around 2.3-fold increase in’ to clarify the magnitude of the association.

*Thank you for this helpful recommendation. We have removed the statistical values from the	main sentence and revised the phrasing to emphasize the magnitude of the association, in line	with both your suggestion and prior reviewer preferences.*

*Page 7, lines 12-17: “Post-transplant ECMO use of greater than 8 days was associated with	approximately a 2.3-fold increase in the odds of developing invasive candidiasis. Other clinical	factors, including antifungal prophylaxis in the 90 days prior to IC, CMV disease, diabetes	mellitus, high comorbidity burden, bilateral lung transplant, and pre-transplant steroid use, were	not meaningfully associated with increased risk. No individual indication for transplant was	associated with a greater likelihood of IC. Full results are provided in Table 3.”*

\# Please clarify this phrase “No single indication for transplant increased the odds of IC”.

*Thank you for pointing this out. We have revised the sentence to clarify that no primary	transplant indication—such as COPD, ILD, or IPF—was associated with increased odds of	developing invasive candidiasis in our multivariable analysis.*

*Page 7, lines 15-17: “None of the primary indications for lung transplant (e.g., COPD, ILD, IPF)	were associated with increased odds of developing invasive candidiasis.”*

\# Please note that the first two sentences in the Effect of Invasive Candidiasis on Mortality part are not appropriate for this part.

*Thank you for this observation. While these sentences describe matched cohort characteristics,	we believe they provide important context for interpreting the mortality outcomes. We have	revised the paragraph to clarify that this information pertains specifically to the matched cohort	used for survival analysis.*

*Page 7, lines, 21-26: “To evaluate the impact of invasive candidiasis on survival, we matched	126 lung transplant recipients with IC to 252 without IC (Figure 2). Table 4 summarizes the	baseline characteristics and covariate balance of the matched cohort. The median follow-up after	the index date was 439.0 days (IQR: 770.0 days), with 97 deaths (25.7%) observed during the	follow-up period. Median time to death was 347.0 days (IQR: 703.0 days). Lung transplant	recipients with IC had a higher mortality rate compared to matched recipients without IC, with	an adjusted hazard ratio of 2.31 (95% CI: 1.45 to 3.67), reflecting more than a twofold increase	in the risk of death.”*

* Please ensure that Table 4 is adequately interpreted, as its interpretation is even more important than that of Table 3.

*Thank you for this important point. We agree that proper interpretation of Table 4 is essential for	understanding the validity of the mortality analysis. We have added a sentence to the Results	section to highlight that the baseline characteristics between the IC and non-IC groups were well	balanced after matching, with standardized differences indicating minimal residual confounding.*

*Page 7, lines 22-25: “As shown in* ***Table 4****, baseline characteristics between recipients with and	without IC were well balanced after matching, with standardized differences falling below the	commonly accepted threshold of 0.1, indicating good covariate balance across groups.”*

* Please provide appropriate titles for both figures, as they currently lack titles.

*Thank you for this helpful suggestion. We have added informative titles to both figures to better	reflect the content and support interpretation.*

\# Regarding Table 3, please note that it is unnecessary to include SE and Z values. It is recommended to remove these values from the table.

*Thank you for this suggestion. We agree that the standard error and Z-statistic columns are not	essential for interpretation and have removed them from Table 3 to streamline presentation and	focus on the most clinically relevant information.*

* Ensure that consistent variable names are used across all tables and carefully review them for uniformity. Additionally, clarify the rationale for including certain variables in Table 4 that are absent from Table 3 (e.g., COPD/Bronchiectasis).

*Thank you for this observation. We have reviewed all tables to ensure consistent variable naming	and formatting. In response to your question regarding variable selection, Table 4 reflects the full	set of baseline characteristics used to assess covariate balance after matching for the mortality	analysis. In contrast, Table 3 presents variables included in the multivariable logistic regression	model to assess risk factors for invasive candidiasis. As such, some variables (e.g.,	COPD/Bronchiectasis) were relevant for matching and descriptive purposes but were not	selected	for the regression model, which focused on variables selected a priori based on clinical	relevance and existing literature.*

Discussion section
* Please note that this section currently requires further elaboration from literature. It is recommended to include a more detailed comparison of your study with previous research and, if possible, please discuss the reasons for any discrepancies, which could be attributed to for example, different methodologies.

*Thank you for this helpful suggestion. We have expanded the Discussion to include a more	detailed comparison with prior studies. We now highlight both concordant findings and potential	reasons for differences, including variation in study design, population size, timing and	definitions of IC, and antifungal prophylaxis practices.*

*Page 7, lines 36-50: “Invasive candidiasis occurred in approximately 10.2% of LTRs in our national	cohort, with a median time to diagnosis of 32 days. This finding is consistent with a prior single-	center study by Baker et al., which reported an IC prevalence of 11.4% and a similar median time	to diagnosis of 31 days(7). In contrast, Marinelli et al. described a lower prevalence of 3.5%;	however, their study only evaluated episodes of candidemia and did not include other forms of	invasive Candida infections, such as surgical site or pleural space infections(8). This difference	highlights the importance of case definition in determining incidence estimates. Our study used	administrative billing codes, which allow for large-scale cohort identification but may include	both candidemia and deep-seated non-bloodstream infections. The broader definition used in	our claims-based approach may explain the higher observed prevalence compared to studies	that only included microbiologically confirmed bloodstream infections. Furthermore, differences	in antifungal prophylaxis practices—such as the use of inhaled amphotericin B versus systemic	triazoles—may have contributed to variability in observed rates across studies. These findings	align with prior large-scale surveillance data from the Transplant-Associated Infection	Surveillance Network (TRANSNET), which identified Candida species as the most common cause	of invasive fungal infections across solid organ transplant recipients(3). Our findings build on this	by focusing specifically on lung transplant recipients and demonstrating that IC remains a	common and clinically important complication, even in the context of widespread antifungal	exposure.”*

* Regarding the sentence: “We decided to focus on ECMO support as a potential risk factor by setting the index date ...”, please clarify the rationale for selecting ECMO support.

*Thank you for this suggestion. We have revised the sentence to clarify that ECMO was selected	as a focal risk factor due to its established association with invasive fungal infections in critically	ill and transplant populations, and the availability of a clear temporal anchor in our dataset to	establish chronology. This allowed us to preserve temporal alignment between exposure and	outcome in the multivariable model.*

*Page 8, lines 1-7: “We decided to focus on ECMO support as a potential risk factor because of its	established association with invasive fungal infections in transplant and critically ill populations,	and because the timing of ECMO initiation provided a reliable anchor to establish chronological	directionality between exposure and development of IC.”*

\# Please note that the sentence “We cannot glean ECMO configuration placement position from our dataset. It is likely that femoral lines portend a higher risk of IC than ...” presents a claim without sufficient scientific support. It is recommended to revise and integrate the following phrase: ‘Our dataset does not include information on ECMO cannulation sites. While femoral access may increase the risk of IC, further studies are needed to confirm this association’.

*Thank you for this thoughtful comment. We have revised the sentence to remove unsupported	assumptions and incorporated the reviewer’s suggested language to better reflect the limitations	of our data and the need for further research.*

*Page 8, lines 9-12: Our dataset does not include information on ECMO cannulation sites. While	femoral access may Increase the risk of invasive candidiasis due to higher potential for	contamination and vascular complications, further studies are needed to confirm this	association.*

* Regarding your sentence: “We found that patients with IC have more than twice the probability of mortality compared to their matched partners”. I did not find the corresponding results in the previous section.
*Thank you for this helpful observation. We have clarified this sentence in the Discussion to	ensure it directly reflects the hazard ratio reported in the Results section and aligns with the	language used earlier in the manuscript.*

*Page 8, lines 26-29: “We found that lung transplant recipients with invasive candidiasis had a	more than twofold increased risk of death compared to matched recipients without IC,	consistent with the adjusted hazard ratio of 2.31 reported in our Cox proportional hazards	model.”*

**Reviewer #7:**

 A very comprehensive study. Data are available, so it is a nice addition to the potential resources made available to others researching similar topics. It is a "complex read" but that is very much essential here.

*We thank Reviewer #7 for their thoughtful and encouraging feedback. We appreciate your	recognition of the study’s comprehensiveness and the potential utility of the dataset for future	research. We are grateful for your supportive comments.*

I noted a misspell [Invasive Candidiasis. . . . 1 paragraph, last line, "diagnositic"(sic)] -- (Can't believe I saw it however, due to the 'density' of this writing). I like the detail oriented approach even for the following: implementation of Elixhauser over Charlton Comorbidity Scoring--that makes sense with, provides and example for, a study focused on Labs data.

*Thank you for your close and thoughtful review. We have corrected the typographical error	noted in the Discussion. We also appreciate your positive feedback on the use of the Elixhauser	Comorbidity Index, which we felt was well-suited to this claims-based study.*

Once again, we would like to express our gratitude to the reviewers for their valuable feedback, which has been instrumental in refining our manuscript. We trust that the revisions made in response to their comments have addressed the concerns raised and improved the overall quality of the paper. We look forward to your further feedback and hope that the revised manuscript is now suitable for publication. Thank you for your consideration and for the opportunity to resubmit our work.

Sincrerely,

Kelly M. Pennington, MD
