# Supplementary material for: Invasive candidiasis following lung transplant: An Assessment of impact utilizing a national insurance claims cohort
Source: PLoS One. 2025 Aug 21;20(8):e0330162. doi: 10.1371/journal.pone.0330162 (PMC12370120; doi:10.1371/journal.pone.0330162)
Supplement: Attachment — Submitted filename: Letter_to_Reviewers_auresp_2.docx [file pone.0330162.s005.docx]

Dear Editorial Team:

We thank the editorial team and all reviewers for their time and detailed evaluation of our manuscript, *“Invasive Candidiasis Following Lung Transplant: An Assessment of Impact Utilizing a National Insurance Claims Cohort.”*

**Reviewer #1:**

The manuscript reviewed by 6 reviewer and revised as my previous comments. It is suitable for publication.

*We sincerely thank the reviewer for their positive feedback and continued support of our manuscript.*

**Reviewer #2:**

Thank you for addressing my comments. Although I would prefer to see the mortality modeled with IC as a time-dependent covariate, the choice of a psudo index date is not unheard of in statistical modeling. The manuscript is much improved with better clarity.

*We appreciate the reviewer’s thoughtful feedback and are grateful for the recognition of the improvements made to the manuscript. We acknowledge that modeling IC as a time-dependent covariate is a valid and elegant alternative. In this analysis, we selected a pseudo index date approach to align with our dataset structure and facilitate matching on temporal proximity and hospital length of stay. We agree that future work could benefit from incorporating time-dependent modeling to validate and extend our findings.*

**Reviewer #4:**

The authors present a well-conducted and clinically relevant retrospective cohort study that addresses a critical and underexplored aspect of post-lung transplantation care. The use of a large national insurance claims database provides robust real-world evidence on the incidence and impact of invasive candidiasis (IC) in lung transplant recipients. The methodology is sound. The manuscript is clearly written, the results are compelling, and the conclusions are well-supported by the data. I recommend acceptance of this article.

*We thank the reviewer for their positive feedback and thoughtful assessment of our study. We greatly appreciate the recommendation for acceptance.*

**Reviewer #5:**

Thank you for answering all my questions and changing the manuscript accordingly. There is one last thing that should be formulated differently:

The authors explain (both in "my" answer and in one for reviewer 2) that they chose the variables for the regression model based on clinical knowledge and literature, but they state that age was excluded "due to collinearity with other factors and lack of a significant univariable association". But exactly significance or non-significance in a univariable model should not be used to decide a bout including a variable, because the p-value can change considerably as soon as additional variables are taken into account.

Therefore, the authors should delete this part of the sentence. If there indeed is strong (multi-)collinearity, this is reason enough to not include age. If, however, the collinearity is not very strong, I suggest to include age in the model as an additional covariate. In any case, it has to be explored and explained properly.

*We thank the reviewer for this important and insightful comment. We agree that variable selection should not rely on statistical significance in univariable models, and we appreciate the opportunity to clarify our rationale.*

*In our analysis, age demonstrated moderate collinearity with comorbidity burden (Elixhauser score), and including both in the multivariable model introduced instability in the model estimates. Given that comorbidity burden was more strongly associated with our outcome of interest and is clinically relevant in this context, we prioritized its inclusion over age. We have revised the manuscript to reflect this clarification and have removed the reference to univariable significance. The updated text now reads:*

*“Age was evaluated but excluded from the final model due to collinearity with comorbidity burden, which was prioritized given its stronger association with IC and greater clinical relevance.”*

**Reviewer #6:**

 I sincerely thank the authors for their thoughtful and thorough revisions to the manuscript. Almost all of my previous comments have been addressed. Before resubmitting, I recommend reviewing the revised version without Track Changes to ensure there are no minor issues, such as the presence of two periods at the end of the conclusion sentence in the abstract.

*We thank the reviewer for their careful review and kind comments. We have removed the extraneous period in the abstract and carefully reviewed the revised manuscript for any remaining formatting or typographical errors before resubmission.*

Also, please note that the code “112.4” appears twice in the following sentence: “In our population, ... for IC (ICD-9: 112.4, 112.5, 112.8, 112.4, 112.83; ICD-10: 16 B37.1, B37.5, B37.6, B37.7) in ... procedure”.

*This has been corrected in the revised version, and the duplicate* ***112.4*** *code has been removed.*

Additionally, I appreciate your attention to comment “Since your study follows a cohort design, it is preferable not to use the terms “Case” and “Control”, which are more relevant to case-control studies. Please revise these terms throughout the text and figures, replacing them with ‘diseased’ and ‘non-diseased’, respectively. Additionally, when referring to mortality outcomes, please use ‘exposed’ and ‘unexposed’ instead”. However, a few instances of these terms still appear in the text and should preferably be corrected.

*Thank you for this important clarification and for previously highlighting the need to align terminology with cohort study design conventions. We carefully reviewed the full manuscript and have now revised all remaining instances of the terms “case” and “control.” Specifically, we have replaced “case”/“control” with “diseased”/“non-diseased” in sections referring to IC status, and with “exposed”/“unexposed” when referring to matched groups in the mortality analysis. Figure titles and flowchart labels have been updated accordingly. We appreciate the reviewer’s close reading and attention to methodological precision.*

Once again, we would like to express our gratitude to the reviewers for their valuable feedback, which has been instrumental in refining our manuscript. Thank you for your consideration and for the opportunity to resubmit our work.

Sincrerely,

Kelly M. Pennington, MD
